# The impact of basketball on the physical health of Chinese middle school students aged 12–18: a systematic review and meta-analysis

**DOI:** 10.3389/fpubh.2025.1692668

**Published:** 2025-12-19

**Authors:** Sun Richen, Xu Xiaolong, Lin Weilong, Sun Xiaoning, Wang Hengtong

**Affiliations:** School of Physical Education, Guangzhou Sport University, Guangzhou, China

**Keywords:** basketball, secondary school pupils, physical fitness, meta-analysis, health

## Abstract

**Purpose:**

This systematic review evaluates the impact of basketball on the physical fitness of Chinese middle school students, providing evidence-based support for research on how basketball enhances the physical health of Chinese students aged 12–18.

**Method:**

Using CNKI, WanFang, WeiPu, PubMed, Web of Science, Embase, and Cochrane as search databases, the search period spanned from the inception of each database to March 4, 2025. Literature screening was conducted using the PICOST model. Ultimately, nine publications comprising 16 studies were included. Quality assessment was conducted using the PRISMA statement and the Cochrane Handbook for Systematic Reviews of Interventions. Statistical analysis and publication bias testing were performed using Review Manager 5.4 and Stata 17 software.

**Result:**

The study included 736 secondary school students, comprising 387 in the experimental group and 349 in the control group. Meta-analysis findings indicate that basketball positively impacts secondary school pupils' physical fitness, with statistically significant outcomes. However, effects vary across different fitness components. Basketball demonstrated statistically significant effects on 50 m sprint performance [MD = −0.41 s, 95% CI (−0.45, −0.36), *P* < 0.00001]. Basketball training also produced statistically significant effects on sit-and-reach flexibility [MD = 2.22 cm, 95% CI (1.02, 3.41), *P* = 0.0003 < 0.05]. Basketball participation yielded statistically significant effects on middle school students' standing long jump performance [MD = 4.18 cm, 95% CI (2.56, 5.79), *P* < 0.00001]. Basketball participation had statistically significant effects on middle school students' sit-up performance [MD = 4.58 t, 95% CI (2.66, 6.50), *P* < 0.00001]. Basketball participation had a statistically significant effect on secondary school students' 1,000 m performance [MD = −11.70 s, 95% CI (−20.00, −3.39), *P* = 0.006 < 0.05]. Basketball participation had a statistically significant effect on secondary school pupils' 800 m performance [MD = −10.59 s, 95% CI (−15.46, −5.72), *P* < 0.0001]. However, basketball participation did not yield statistically significant effects on pull-up performance.

**Conclusion:**

Basketball training yields comprehensive benefits for enhancing the physical fitness of secondary school students, with improvements primarily observed in speed, flexibility, strength, and endurance. However, no significant enhancement in upper-body strength was noted. The extent of improvement varied according to gender, year group, and duration of the intervention programme.

**Systematic review registration:**

PROSPERO, identifier CRD420251022157.

## 1 Introduction

Currently, the decline in physical fitness among adolescents worldwide has become a serious public health challenge. The World Health Organization (WHO) has consistently warned that over 80% of adolescents worldwide fail to meet its recommended standard of at least 60 min of moderate-to-vigorous physical activity daily. This phenomenon has led to a series of health issues, including rising rates of overweight and obesity, declining cardiorespiratory fitness, insufficient muscle strength, and persistently high rates of myopia. These problems pose long-term threats to individuals' future health trajectories and the overall societal burden of disease. Middle school students are in adolescence, a critical transition period from childhood to adulthood. This stage represents not only a “golden window” of rapid physical development and peak physiological plasticity but also a pivotal time for establishing lifelong habits and behavioral patterns. Therefore, implementing effective health interventions during this phase—particularly enhancing physical fitness through increased physical activity—holds crucial strategic importance. Physical exercise, as a structured and planned form of bodily activity, is widely recognized as the most effective and economical means to counteract physical health issues among adolescents. Among various sports, basketball demonstrates immense potential for promoting adolescent physical health due to its unique attributes and global popularity. Basketball is not only a sport widely favored by adolescents, but its inherent movement patterns also align closely with the demands of physical health development. It is a comprehensive full-body exercise integrating high-intensity intermittent sprints (anaerobic exercise), sustained running (aerobic exercise), jumping, throwing, and physical contact. This allows for the simultaneous development of multiple physical qualities including speed, strength, agility, coordination, and endurance (1). Furthermore, as a team sport, basketball cultivates teamwork, communication skills, and decision-making abilities among adolescents, positively contributing to their psychosocial development. Some studies indicate that long-term basketball training not only improves physical fitness but also enhances participants' functional capacity and overall quality of life. Therefore, integrating basketball as a core component of school physical education or promoting it through extracurricular programs represents an attractive and feasible pathway to enhance the physical health of middle school students. Academic circles have extensively explored the health benefits of basketball. Wei (2) noted in their research that basketball significantly positively impacts students' physical fitness and mental health, strongly recommending schools enhance basketball participation through measures like expanding basketball clubs and improving facilities (3). This perspective aligns with numerous international studies. Research conclusions are relatively consistent regarding strength, speed, and agility. For instance, a 2024 study on adolescent basketball players found that Complex Training significantly improved endurance, speed, power, and agility (4). An earlier study also confirmed that a 4-week plyometric training program effectively enhanced basketball players' speed, agility, and lower-body muscle strength (5). Shengnan and Jing (6) also observed improvements in upper-body strength through “Little Basketball,” though the study did not explicitly address developmental stage differences. Collectively, these studies demonstrate that basketball's frequent sprints, direction changes, jumps, and shots effectively stimulate the neuromuscular system, promoting strength, speed, and agility development. A 15-week basketball training program similarly observed significant improvements in female students' sprint speed and agility (7). The positive impact of basketball on cardiorespiratory endurance is also widely recognized. The continuous full-court running and high-intensity transitions between offense and defense in basketball provide excellent cardiovascular training. Multiple studies indicate that regular basketball training significantly increases adolescents' maximal oxygen uptake (VO_2_max), the gold standard for measuring cardiorespiratory endurance (8–14). One study even explored the positive relationship between aerobic capacity and agility, as well as core muscular endurance, suggesting the foundational role of cardiorespiratory fitness in overall physical fitness (15). However, regarding body composition and flexibility, existing research findings remain somewhat controversial and uncertain. Yanlei (16) noted in his meta-analysis that basketball can moderately improve adolescent body composition, though the effect is not pronounced. He also found no significant enhancement in flexibility from basketball training (16). This finding aligns with some international studies, where conflicting reports exist regarding basketball training's effects on body composition and blood pressure, with inconsistent outcomes on flexibility improvement (17). This discrepancy may stem from basketball's technical movements predominantly involving explosive contractions of large muscle groups, with limited emphasis on extensive joint stretching. In contrast, Chen Deyi (49) concluded that long-term basketball participation significantly enhances speed, strength, endurance, agility, and “flexibility” in senior high school male students. This divergence in conclusions underscores the need for deeper, more systematic research on basketball's impact on specific physical attributes, particularly flexibility (9). While existing research offers valuable insights into basketball's impact on adolescent health, several notable gaps remain: inconsistent findings: as noted earlier, conclusions regarding basketball's effects on body composition and flexibility vary significantly, even contradicting each other. Lack of comparative systematic reviews: while numerous individual studies exist on basketball's effects, few systematic reviews or meta-analyses directly compare basketball with other team sports in terms of improving overall health indicators among middle school students (10, 18–26). Generalizability concerns: many studies sample “youth basketball athletes,” who may be selected individuals with training intensities and systematic approaches far exceeding those of typical middle school students. Thus, the direct applicability of these findings to school-based, universal basketball programs for all students remains questionable (27–29). Insufficient quantification of effects: most studies provide qualitative conclusions or single effect values, lacking a comprehensive, quantitative evidence system to precisely describe the average effect size of basketball on various physical health indicators among secondary school students. To address these research gaps, this study aims to employ a meta-analysis methodology to systematically retrieve, screen, and synthesize quantitative research from domestic and international sources on the impact of basketball interventions on the physical health of secondary school students. This study will strictly adhere to systematic review inclusion and exclusion criteria, extract data from qualifying literature, conduct quality assessments, and ultimately combine effect sizes using statistical methods. Through this meta-analysis, this study aims to provide educators, sports policymakers, and parents with more comprehensive and reliable scientific evidence regarding the health benefits of basketball. This will offer a clear and feasible practical pathway for optimizing school physical education curriculum design, promoting youth sports activities, and effectively enhancing the physical health levels of secondary school students in China and globally.

## 2 Materials and methods

### 2.1 Research registration

To prevent duplicate research, this study has been prospectively registered with PROSPERO CRD420251022157, ensuring complete consistency between the research methodology and the registered protocol.

### 2.2 Sources of data

#### 2.2.1 Retrieval personnel

The first and second authors conducted the literature search independently and double-blinded, strictly adhering to the PRISMA Guidelines (30). The PRISMA Checklist, included in the Supplementary Data Sheet 1, explicitly outlines the research methodology and all relevant aspects of this review.

#### 2.2.2 Retrieval time

The search includes studies published up to March 4, 2025.

#### 2.2.3 Database

This study was conducted in CNKI, WeiPu, WanFang, PubMed, Embase, Cochrane, and Web of Science.

#### 2.2.4 Search keywords

Search terms: Basketball, Basketballs, Netball, Netballs, Middle school student, middle school students, high school students, physical health, physical health status.

#### 2.2.5 Retrieval strategy

This study conducted a search across the CNKI, WeiPu, WanFang, PubMed, Embase, Cochrane, Web of Science databases. The search was conducted in either Chinese or English, with no restrictions on time or region. The search query was: (((“Basketball”[Mesh]) OR ((((Basketball) OR (Basketballs)) OR (Netball)) OR (Netballs))) AND (((Middle school student) OR (middle school students)) OR (high school students))) AND (((((((((physical health) OR (physical health status)) OR (50-m sprint)) OR (sit-and-reach)) OR (standing long jump)) OR (pull-ups)) OR (one-minute sit-ups)) OR (1000-m run)) OR (800-m run)).

### 2.3 Inclusion and exclusion criteria for literature

The inclusion and exclusion of literature was conducted independently by two authors. The PICOST criteria [P (participants); I (intervention); C (comparison); O (outcome); S (study design); T (time)] were applied (31). In the event of disagreement during the final screening, a third author was consulted to facilitate discussion until consensus was reached. Inclusion criteria required compliance with the following conditions: (a) study population: secondary school pupils, aged 12–18 years; (b) intervention: experimental group received basketball-based intervention (physical education classes, basketball training, extracurricular basketball activities); (c) comparison: control group did not receive basketball-specific intervention; (d) outcome measures: studies must include at least one or more of the following: 50-m sprint, sit-and-reach, standing long jump, pull-ups (males)/1-min sit-ups (females), 1,000-m run (males)/800-m run (females); (e) study design: controlled trials; and (f) intervention duration: the study must clearly specify the intervention period. Studies failing to meet these inclusion criteria will be excluded.

### 2.4 Assessment of literature bias risk

Two independent researchers conducted a bias analysis of the included literature according to the Cochrane Handbook for Systematic Reviews. This primarily encompassed: random sequence generation (whether allocation between the experimental and control groups was fully random), allocation concealment (whether those implementing the basketball intervention were aware of the allocation beforehand), and blinding of participants or personnel (whether basketball intervention practitioners and participants knew their respective group assignments), blinding of outcome assessors (whether outcome assessors knew which group participants belonged, potentially skewing results), incomplete outcome data (whether study results were missing), selective reporting (whether study results omitted test scores or key primary outcomes), and other biases (other factors influencing intervention outcomes). The overall risk assessment of the article is categorized into three levels: (a) high-risk articles: any article containing at least one high-risk item; (b) unknown-risk articles: articles without high-risk items but containing unknown-risk items; and (c) low-risk articles: articles without either high-risk or unknown-risk items.

### 2.5 Extraction of data information

Compile and summarize the basic information of articles included in the analysis, extracting relevant details. Should any data be missing, attempt to contact the authors via email to seek completion of the information. Number the included articles and extract data from the selected literature, primarily comprising: authors, publication year, study subjects, intervention duration, subjects' gender, and sample size. As shown in Table 1 below.

**Table 1:** Basic characteristics of included studies.

**Rank**	**Author**	**Year**	**Object**	**Time (weeks)**	**Gender**		**Sample size**	
					**Male**	**Female**	**Experimental**	**Control**
1	Liu ZW	2022	Junior high school student	12	40	40	40	40
2	Zhou ZG	2020	Junior high school student	36	28		14	14
3	Zhou C	2018	High school student	52	110		57	53
4	Zhang XX	2023	High school student	16	52	28	40	40
5	Zhang XC	2022	Junior high school student	17	64	40	52	52
6	Li HY	2010	High school student	52	53	29	41	41
7	Yang T	2021	Junior high school student	12	64	49	56	57
8	Yang XJ	2010	Junior high school student	52	61	48	72	37
9	Zhao TC	2015	High school student	8	30		15	15

### 2.6 Statistical methods

Statistical analysis was performed using Review Manager 5.4 and Stata 17 software. Since the 50-m sprint, sit-and-reach, standing long jump, pull-ups (male)/1-min sit-ups (female), and 1,000-m run (male)/800-m run (female) were continuous variables, the analysis utilized the mean difference (MD), confidence interval (95% CI), and total effect value *Z*. Heterogeneity among included studies was assessed using the *I*^2^ statistic: *I*^2^ = 0 indicated no heterogeneity; *I*^2^ between 0 and 50% suggested acceptable heterogeneity, warranting a fixed-effects model; *I*^2^ > 50% indicated substantial heterogeneity, requiring a random-effects model (32). The significance level was set at *P* = 0.05, and subgroup analyses were conducted to ensure reliability of results. When more than eight studies are included, a publication bias test is performed.

## 3 Results

### 3.1 Literature search and screening

**Figure 1:**
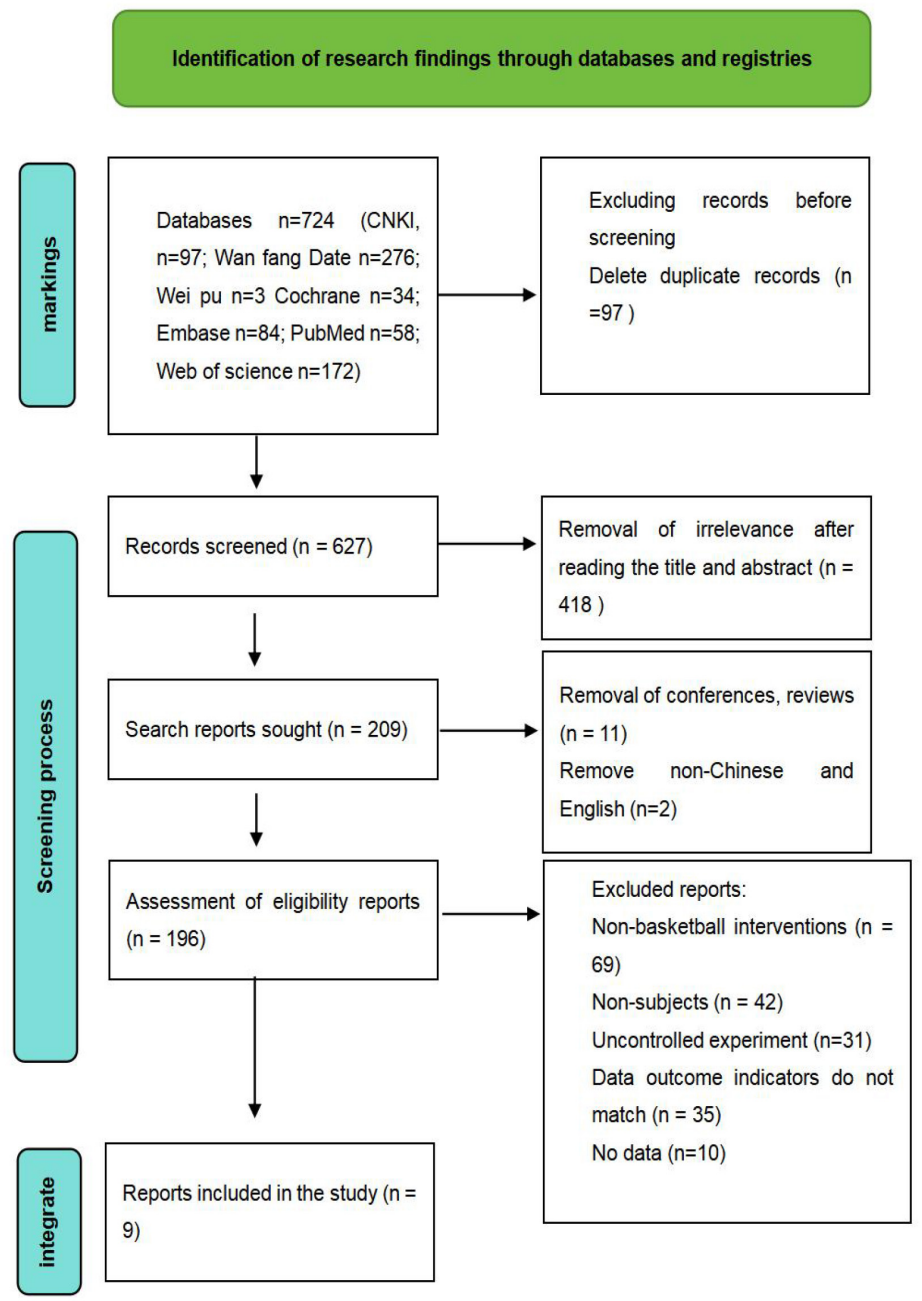
Flow chart of PRISMA literature screening.

Search keywords: basketball, basketball sport, tennis, tennis sport, junior high school students, senior high school students, physical health, physical health status. The databases included: CNKI, WeiPu, WanFang, PubMed, Embase, Cochrane, Web of Science. This search encompassed virtually all relevant literature from the inception of these databases up to 4 March 2025, yielding a total of 724 articles. During the screening process, the retrieved literature was first imported into the EndNote reference management software. Machine screening and manual review identified 97 duplicate articles. Further examination of titles and abstracts revealed that 418 articles were entirely irrelevant to this study. Full-text review revealed 13 conference proceedings or review articles, 69 non-basketball intervention studies, 42 studies not involving secondary school students, 31 non-controlled trials, 35 studies with outcome measures incompatible with this study's criteria, and 10 studies lacking published experimental data. Ultimately, nine studies were included. Figure 1 illustrates the PRISMA screening process. Table 1 presents the basic information of the included studies.

### 3.2 Fundamental characteristics of included literature and evaluation of literature quality

**Figure 2:**
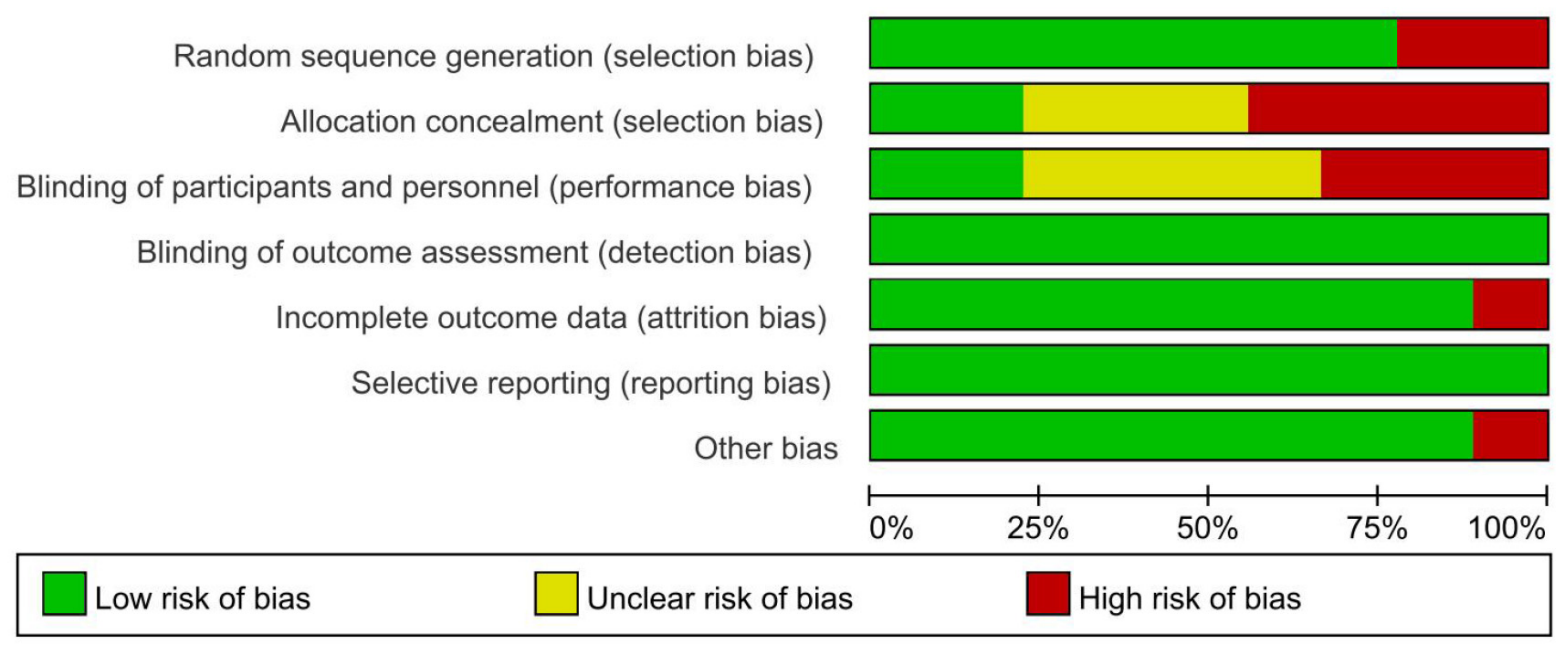
Proportion of each quality evaluation item of included literature.

**Figure 3:**
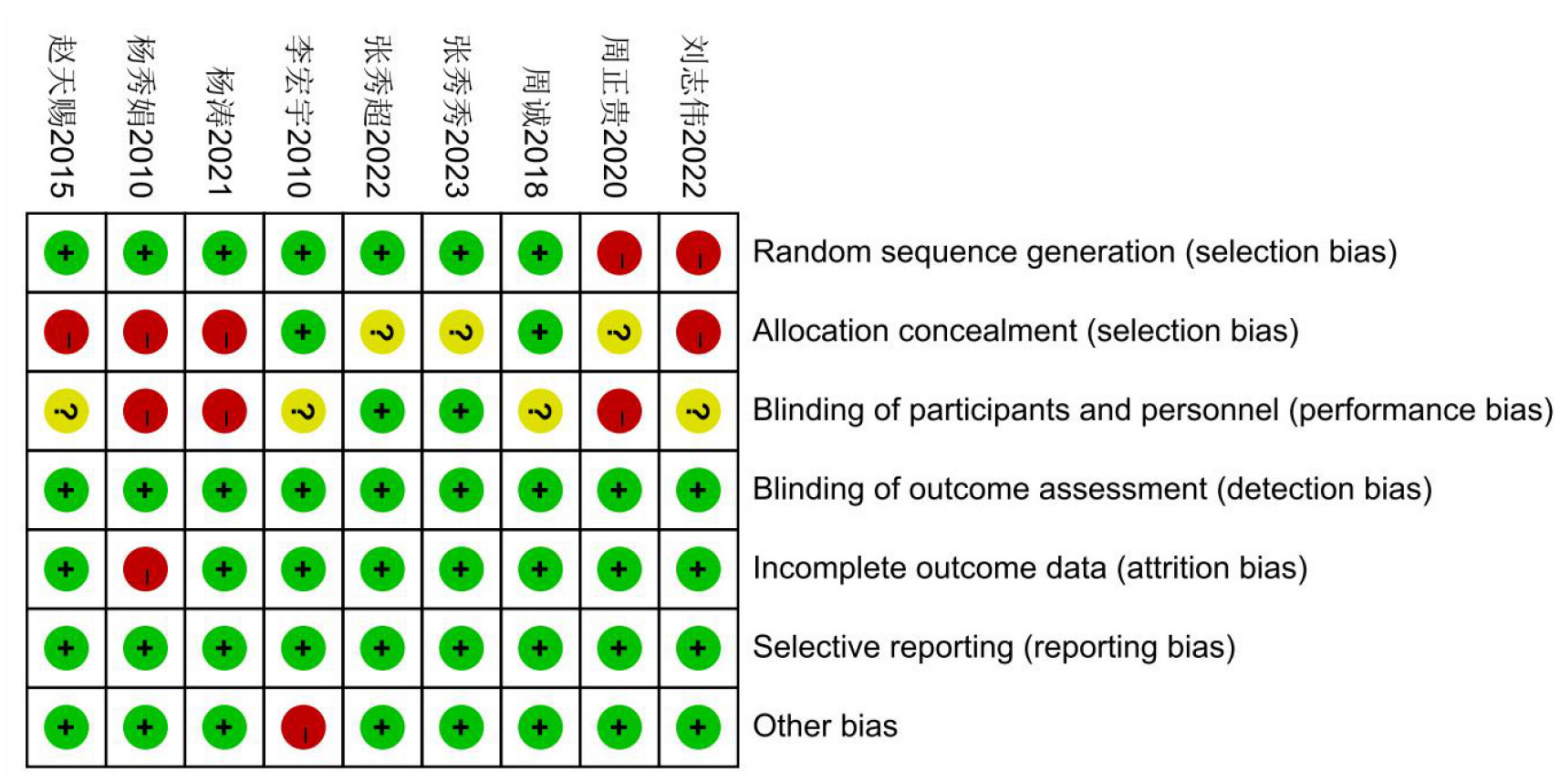
Schematic diagram of quality evaluation of included literature.

**Figure 4:**
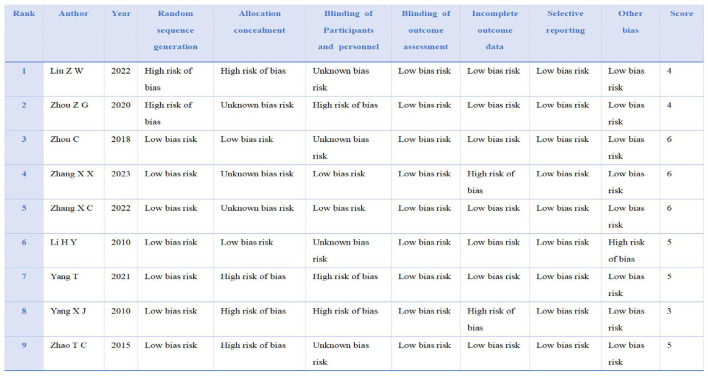
Literature scoring diagram.

The nine articles included in this study comprised: six studies showed high risk of bias, three had unknown risk of bias, and the overall quality of the included literature was low. The nine articles included a total of 762 secondary school pupils: 387 in the experimental groups and 349 in the control groups. Quality analysis of the included articles was conducted using Review Manager 5.4 software to generate a risk of bias plot. As shown in Figures 2, 3. The specific scores for each article are presented in Figure 4.

### 3.3 Meta-analysis results

#### 3.3.1 The impact of basketball on speed qualities among Chinese middle school students

##### 3.3.1.1 Integrity test

**Figure 5:**
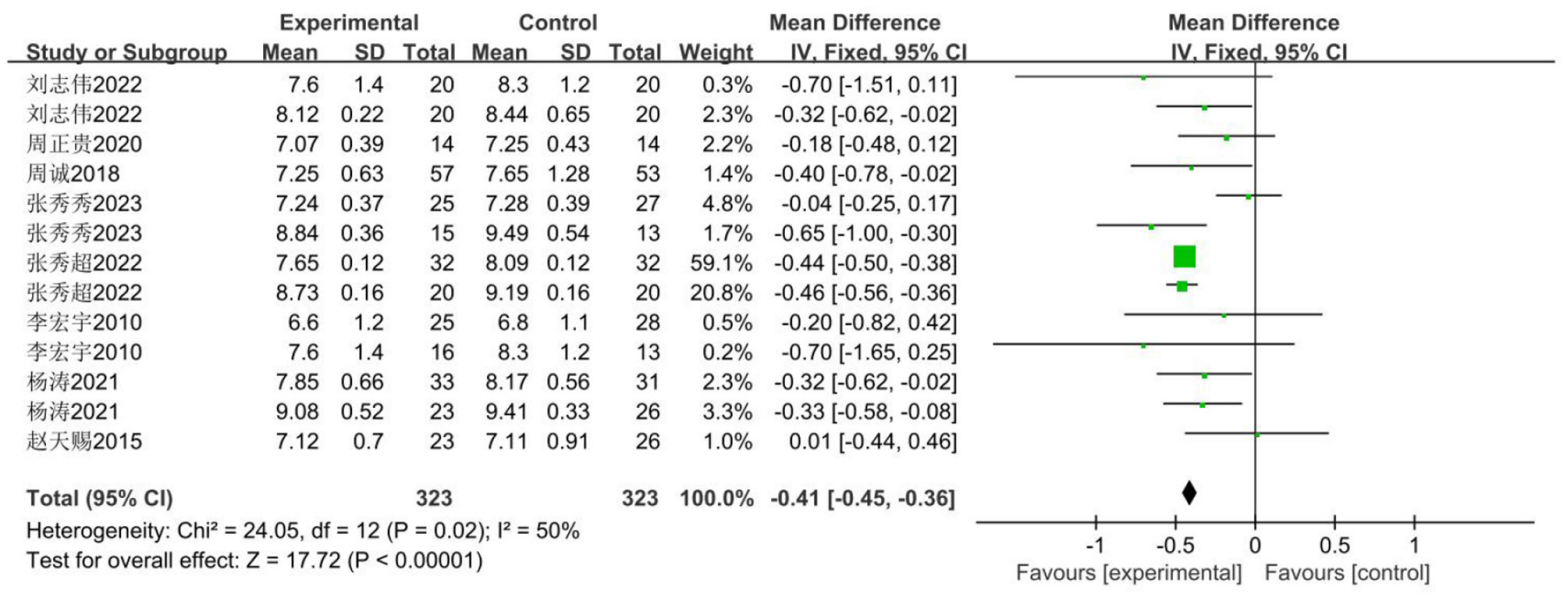
Forest plot of basketball training's impact on 50 m sprint performance in adolescents.

The 50-m sprint time is selected as the evaluation metric for measuring middle school students' speed fitness. Eight publications comprising 14 studies (33–40) were included, examining the impact of basketball training on 50-m sprint performance among secondary school pupils. The total sample comprised 646 pupils, with 323 assigned to the experimental group and 323 to the control group. Heterogeneity analysis revealed moderate heterogeneity among studies (*I*^2^ = 50, *P* < 0.05), necessitating the use of a fixed-effects model for effect estimation. Results are presented in Figure 5. Compared with the control group, the experimental group demonstrated a substantial and statistically significant improvement in 50 m performance [MD = −0.41 s, 95% CI (−0.45, −0.36), *Z* = 17.72, *P* < 0.00001].

##### 3.3.1.2 Publication bias assessment

**Figure 6:**
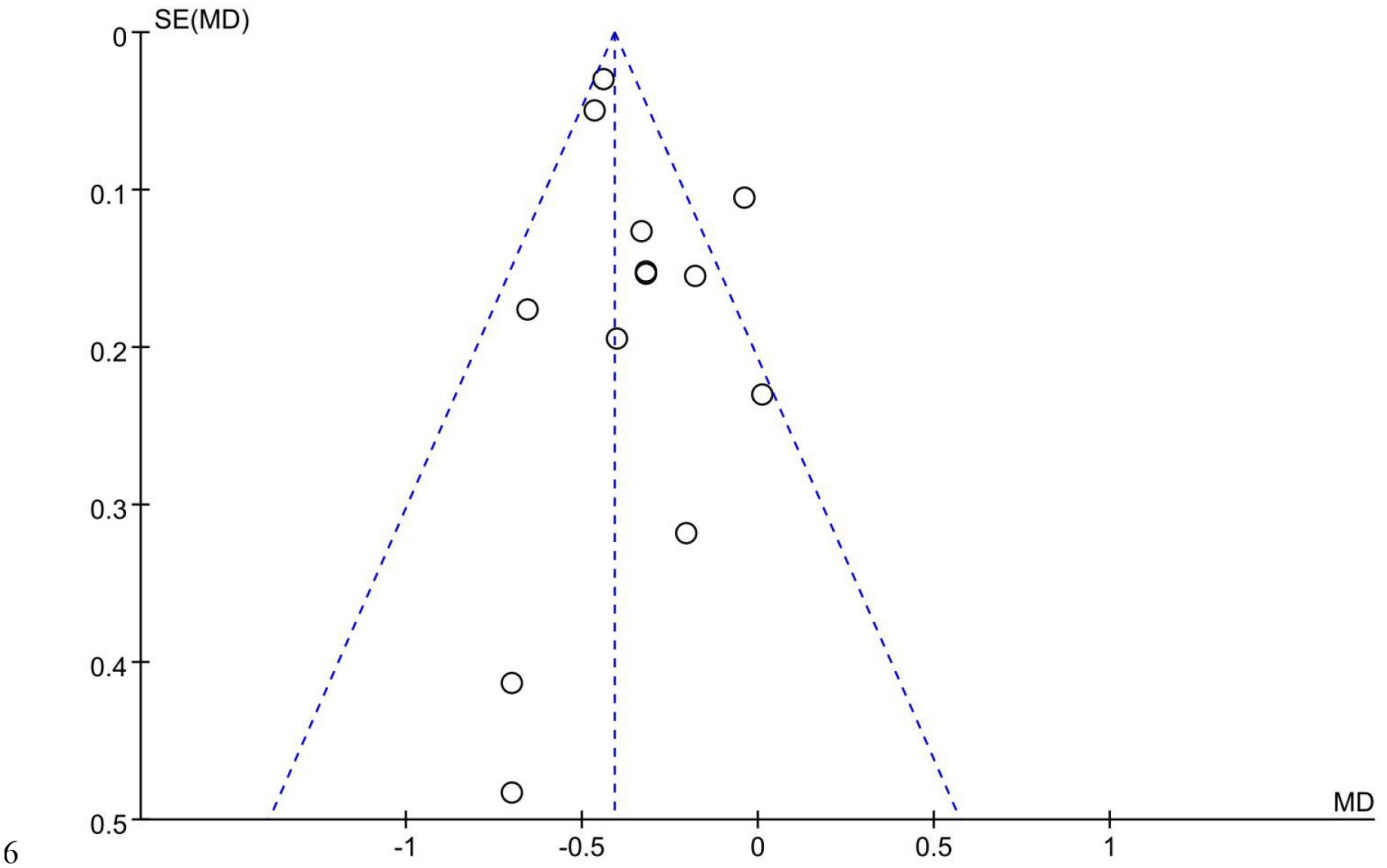
Funnel plot of basketball training's effect on 50 m sprint performance.

The funnel plot (Figure 6) indicates that publications are distributed symmetrically around the mean effect size, suggesting minimal publication bias. Egger's test (*t* = 1.47, *P* = 0.171 > 0.05) further confirms the absence of significant publication bias.

##### 3.3.1.3 Meta-regression and moderator subgroup analysis

Given that the heterogeneity of effect sizes exceeded 50%, meta-analyses were conducted by grouping studies based on gender, intervention duration, and educational stage, as determined by literature characteristics. Results are presented in Table 2, indicating gender as the primary source of heterogeneity. Gender, grade level, and intervention duration were explored as moderating variables to identify key factors influencing basketball's effect on 50 m sprint performance. Regarding gender, females (MD = −0.461 s) outperformed males (MD = −0.390 s). By educational stage, junior secondary students (MD = −0.429 s) demonstrated superiority over senior secondary students (MD = −0.22 s). Concerning intervention duration, programme lasting < 18 weeks (MD = −0.415 s) yielded better outcomes than those ≥18 weeks (MD = −0.280 s).

**Table 2:** Meta-regression analysis of moderating variables on 50 m sprint performance.

**Regulating variable**	**Coefficient of regression**	**Standard error**	***T*-value**	***P* > |*t*|**	**95% CI**
Gender	−0.2046745	0.1131487	−1.70	0.123	−0.4770003, 0.0676513
Year	0.0932886	0.1226802	0.76	0.466	−0.1842333, 0.3708104
Intervention cycle	−0.0365985	0.1553617	−0.24	0.819	−0.3880512, 0.3148542

##### 3.3.1.4 Sensitivity analysis

**Figure 7:**
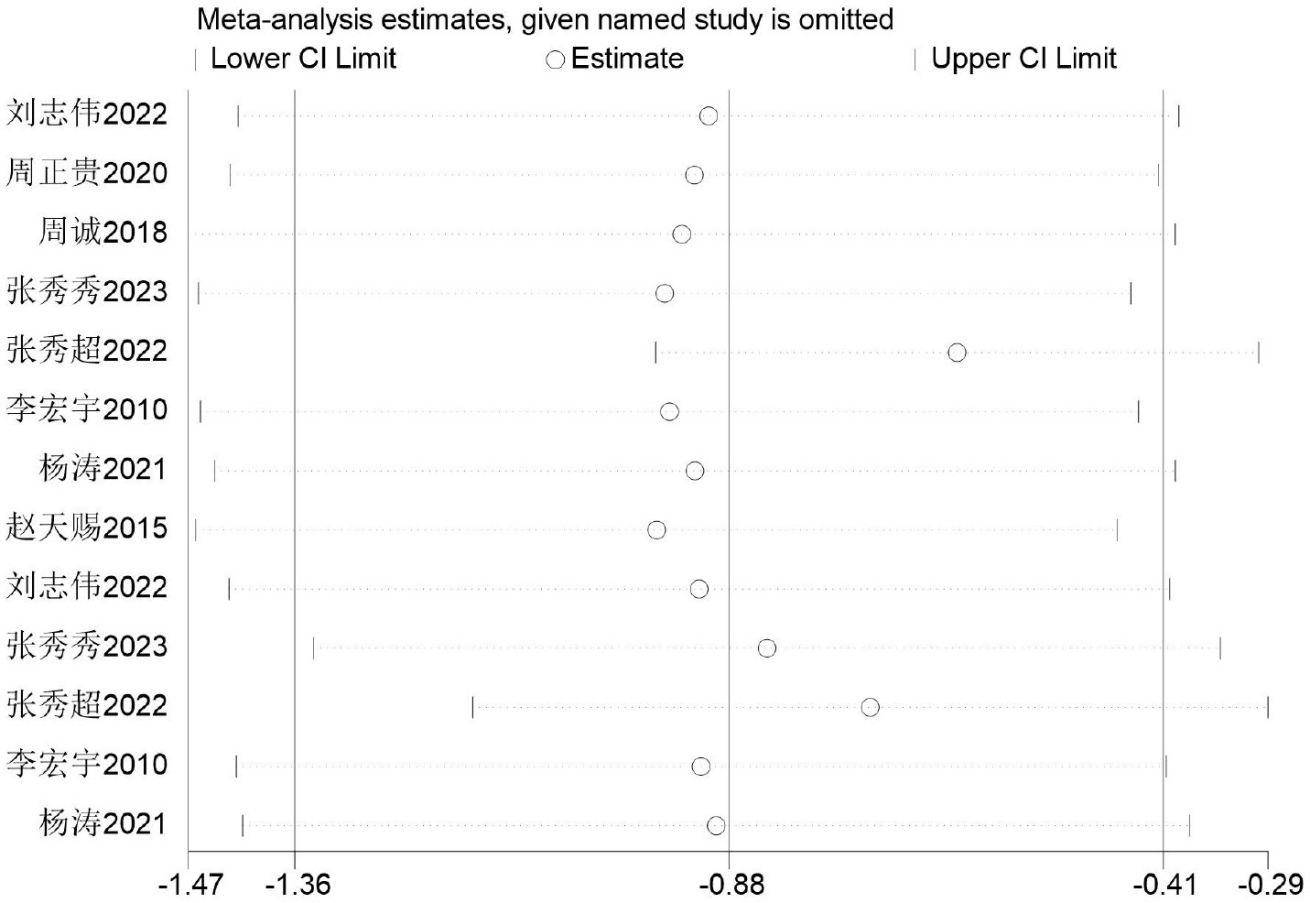
Sensitivity analysis of basketball training's impact on 50 m sprint performance in adolescents.

Firstly, switching between random-effects and fixed-effects models and re-conducting statistical analyses revealed no significant alterations in all statistical outcomes. Secondly, assessing the influence of each study by sequentially excluding one study demonstrated that the pooled effect size MD (0.36 s, 0.43 s) and *I*^2^ ranged from 2 to 54%, with *P* < 0.00001. This indicates the meta-analysis results are stable and reliable (Figure 7).

#### 3.3.2 The impact of basketball on flexibility in Chinese middle school students

##### 3.3.2.1 Integrity check

**Figure 8:**
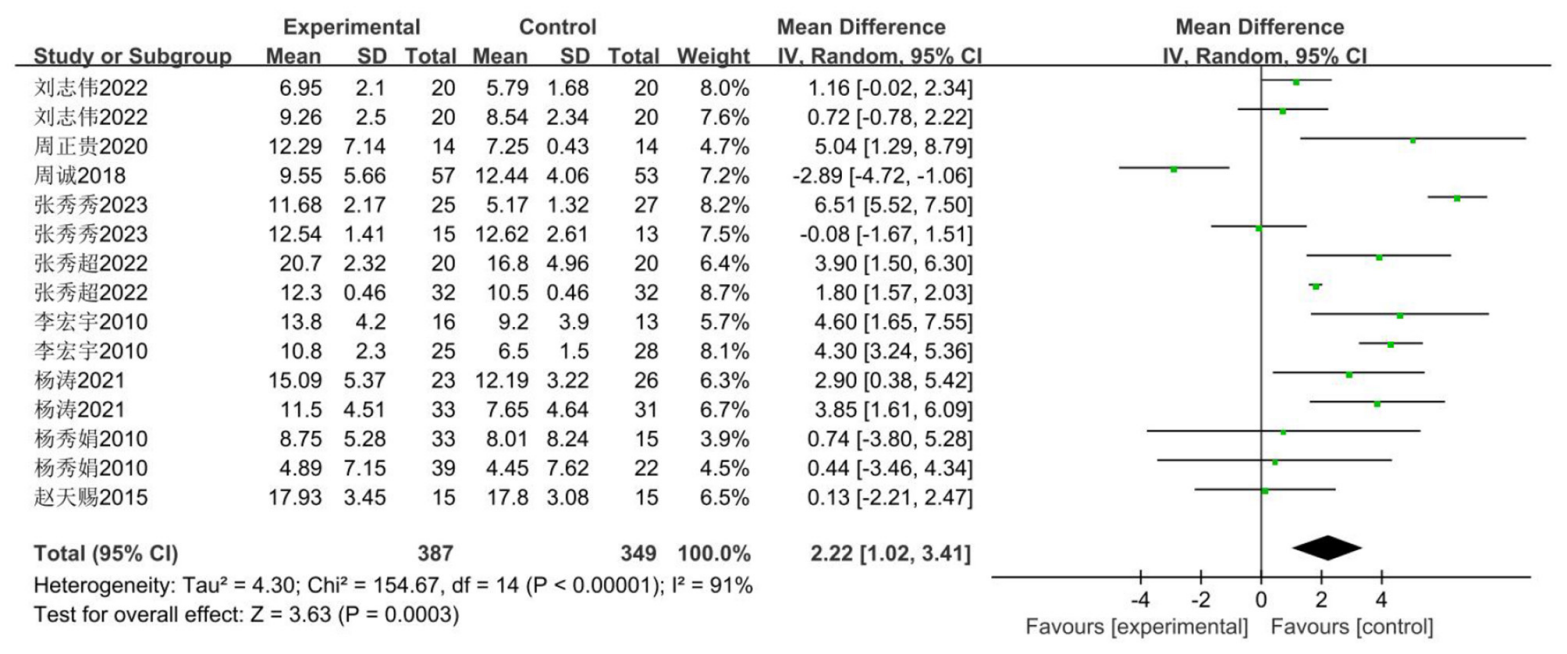
Forest plot of basketball training's impact on sit-and-reach flexibility in adolescents.

The sit-and-reach test is commonly used as an evaluation metric for assessing the physical fitness and health status of middle school students. This paper also employs the sit-and-reach test as its evaluation metric. The impact of basketball training on sit-and-reach performance among secondary school students was examined across nine studies (33–41), comprising 15 investigations involving 736 pupils: 387 in experimental groups and 349 in control groups. Heterogeneity testing revealed substantial heterogeneity (*I*^2^ = 91, *P* < 0.00001). Consequently, a random-effects model was employed for analysis. As illustrated in Figure 8, the intervention group demonstrated a significant improvement in sit-and-reach test scores compared to the control group [MD = 2.22 cm, 95% CI (1.02, 3.41), *P* = 0.0003 < 0.05], indicating statistical significance.

##### 3.3.2.2 Publication bias test

**Figure 9:**
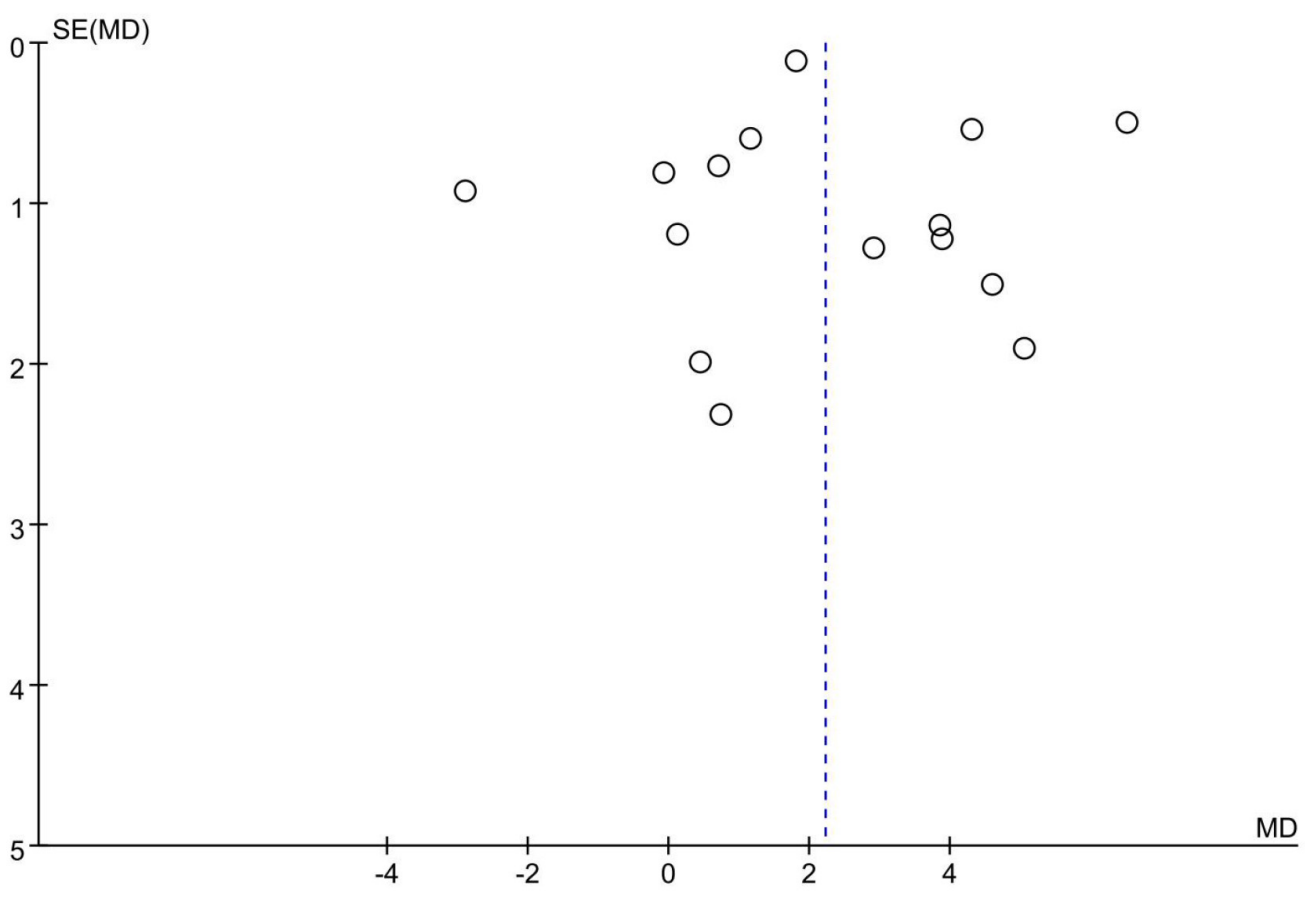
Funnel plot for assessing publication bias in the meta-analysis of basketball training's impact on sit-and-reach flexibility among adolescents.

The funnel plot (Figure 9) indicates that most studies are distributed on both sides of the mean effect, with no apparent publication bias. This conclusion is supported by the results of Egger's test (*t* = 1.90, *P* = 0.08 > 0.05), thus confirming the absence of significant publication bias.

##### 3.3.2.3 Meta-regression and moderator subgroup analysis

Given that the heterogeneity of the meta-analysis exceeded 50%, the meta-analysis was conducted with gender, grade level, and intervention cycle as moderator variables based on the inclusion criteria from the literature. The results are presented in Table 4. Grade level may be a contributing factor to the elevated heterogeneity. Subgroup analyses were conducted based on gender, year group, and intervention cycle, as presented in Table 5. Results indicated that male participants (MD = 2.296 cm) demonstrated superior performance compared to female participants (MD = 1.994 cm) (Table 3); No significant difference was observed between the experimental and control groups in senior secondary school (*P* = 0.164 > 0.05), whereas a significant difference was found in junior secondary school (*P* < 0.0001), with the experimental group achieving markedly higher results than the control group (MD = 1.975 cm); Regarding intervention duration, a significant difference was observed for periods < 18 weeks (*P* = 0.002 < 0.05), with the experimental group showing markedly higher results than the control group (MD = 2.320 cm). No significant difference was found for periods >18 weeks (*P* = 0.209 > 0.05).

**Table 3:** Subgroup analysis of moderating variables on 50 m sprint performance.

**Regulating variable**	**Stratified subgroup**	**Number of studies included**	**MD (95% CI)**	***Z*-value**	***P*-value**
Gender	Male	7	−0.390 s (−0.443, −0.337)	14.51	0.000
	Female	5	−0.461 s (−0.549, −0.373)	10.26	0.000
Year	Junior high school student	7	−0.429 s (−0.477, −0.381)	17.68	0.000
	High school student	7	−0.220 s (−0.365, −0.074)	2.96	0.003
Intervention cycle	< 18 weeks	9	−0.415 s (−0.461, −0.368)	17.58	0.000
	≥18 weeks	5	−0.280 s (−0.496, −0.064)	2.54	0.011

**Table 4:** Meta-regression analysis of moderating variables on sit-and-reach flexibility in adolescents.

**Regulating variable**	**Coefficient of regression**	**Standard error**	***T*-value**	***P* > |*t*|**	**95% CI**
Gender	−0.2418611	1.562887	−0.15	0.880	−3.681752, 3.19803
Year	−0.0951714	1.568679	−0.06	0.953	−3.547811, 3.357469
Intervention cycle	−0.3033198	1.620865	−0.19	0.855	−3.870819, 3.264179

**Table 5:** Subgroup analysis of moderating variables on sit-and-reach flexibility in adolescents.

**Regulating variable**	**Stratified subgroup**	**Number of studies included**	**MD (95% CI)**	***Z*-value**	***P*-value**
Gender	Male	9	2.296 cm (0.627, 3.966)	2.70	0.007
	Female	5	1.994 cm (0.407, 3.581)	2.46	0.014
Year	Junior high school student	9	1.975 cm (1.230, 2.719)	5.20	0.000
	High school student	5	2.114 cm (−0.861, 5.090)	1.39	0.164
Intervention cycle	< 18 weeks	9	2.320 cm (0.878, 3.762)	3.15	0.002
	≥18 weeks	5	2.033 cm (−1.141, 5.207)	1.26	0.209

##### 3.3.2.4 Sensitivity analysis

**Figure 10:**
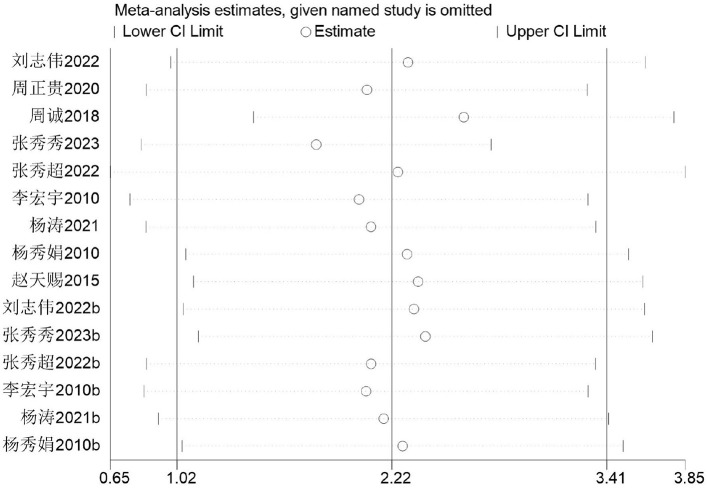
Sensitivity of basketball training to sit-and-reach test results in secondary school students.

Firstly, switching between random-effects and fixed-effects models and re-conducting statistical analyses revealed no significant alterations in all statistical outcomes. Secondly, sequentially excluding individual studies demonstrated that removing any single study had negligible impact on the overall effect size and heterogeneity. The *I*^2^ values ranged from 82 to 92%, and the results remained statistically significant, indicating robust and reliable meta-analysis findings. As shown in Figure 10.

#### 3.3.3 The impact of basketball on lower-body explosive power among Chinese middle school students

##### 3.3.3.1 Integrity check

**Figure 11:**
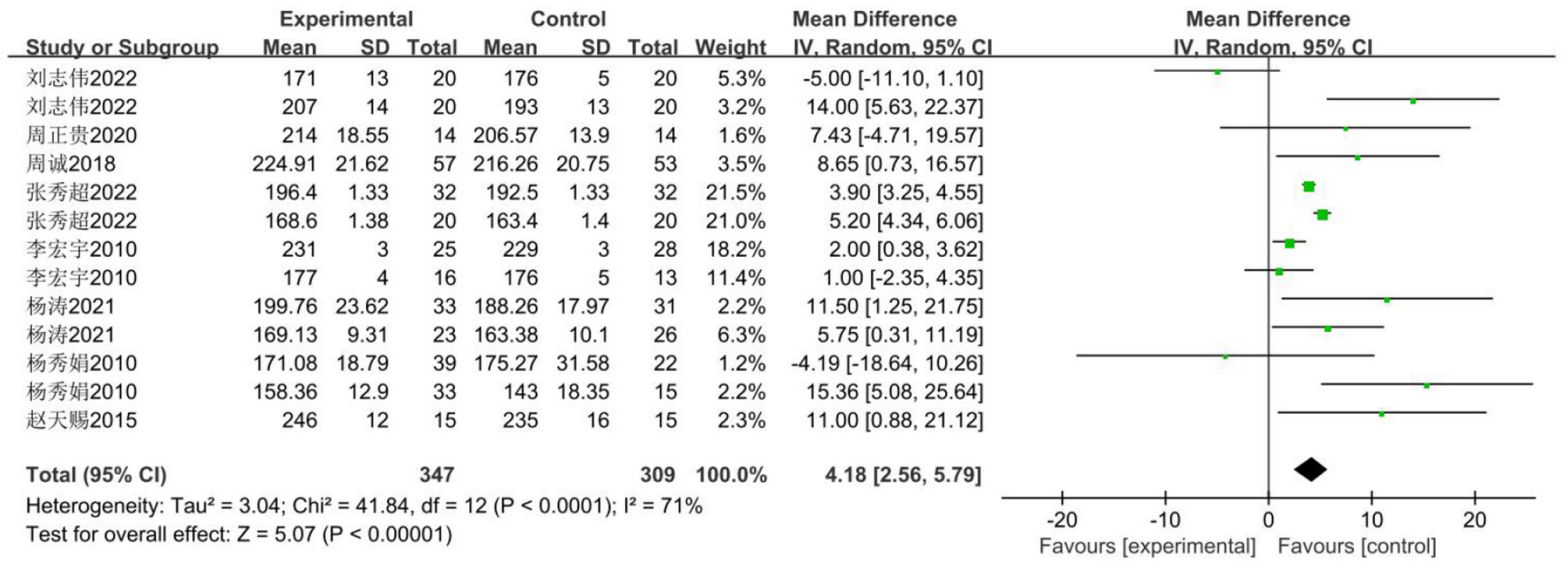
Forest plot illustrating the impact of basketball participation on standing long jump performance among secondary school pupils.

The standing long jump is commonly used as the primary indicator for evaluating lower-body explosive power in Chinese middle school students. The impact of basketball training on standing long jump performance among secondary school students was examined across eight studies (33–41). The 14 studies involved 656 secondary school students, comprising 347 in the experimental group and 309 in the control group. Heterogeneity testing revealed substantial heterogeneity (*I*^2^ = 71%, *P* < 0.0001). Consequently, a random-effects model was employed, with results presented in Figure 11. A significant difference was observed between the control and experimental groups [MD = 4.18 cm, 95% CI (2.56, 5.79), *P* < 0.00001].

##### 3.3.3.2 Bias test

**Figure 12:**
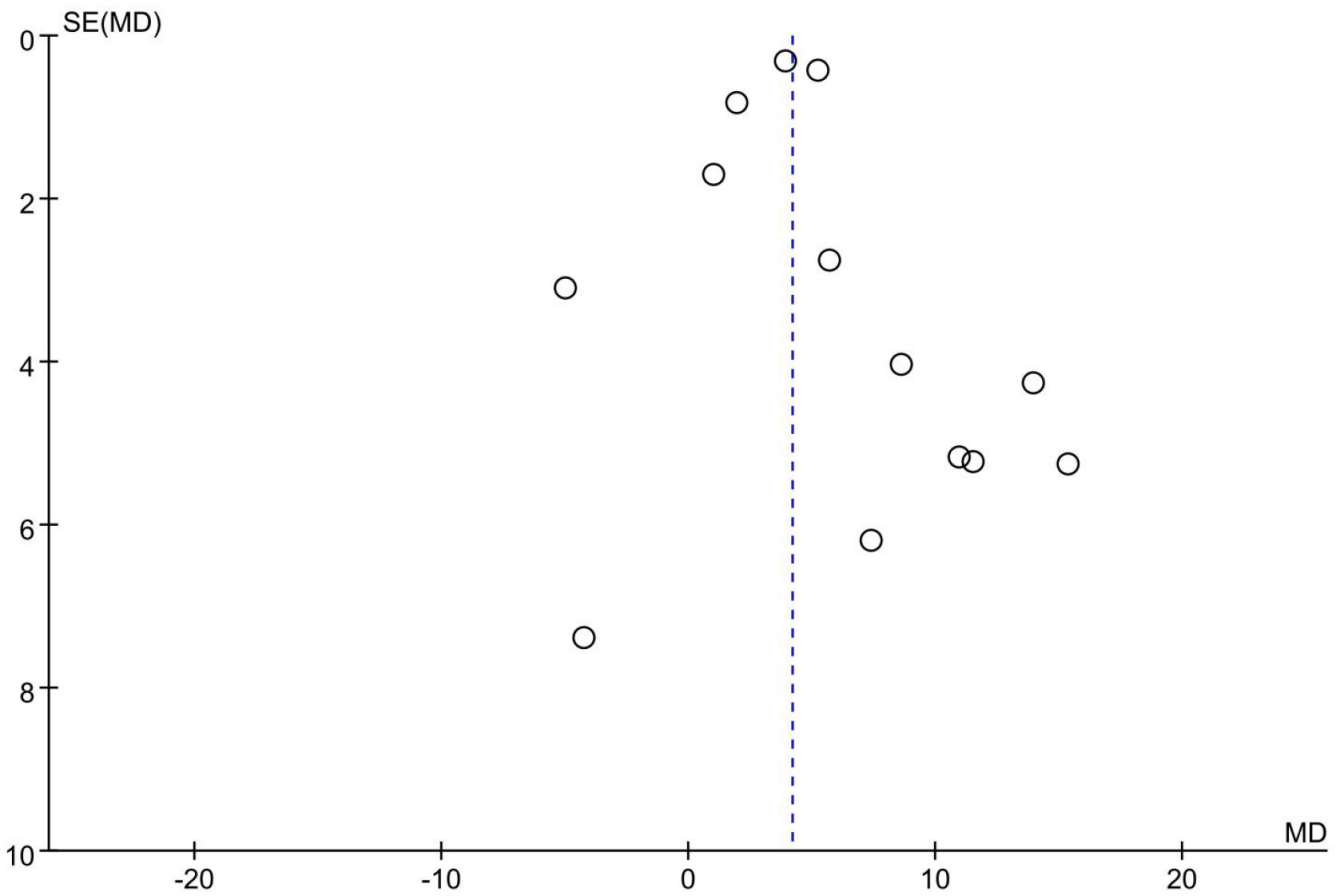
Sensitivity of standing long jump performance in secondary school students to basketball training.

The funnel plot, as shown in Figure 12, indicates that the majority of studies are distributed on either side of the mean effect size. Furthermore, the results of Egger's test indicate that the test statistic is not statistically significant (*t* = 0.46, *P* > |*t*| = 0.645).

##### 3.3.3.3 Meta-regression and moderator subgroup analysis

Given that the heterogeneity of merger benefits exceeded 50%, a meta-analysis was conducted using gender, grade level, and intervention cycle as moderator variables based on the inclusion criteria outlined in the literature. As shown in Table 6, gender may be the primary cause of heterogeneity. Subgroup analyses were conducted with gender, grade level, and intervention cycle as moderating variables. The results are presented in Table 7. Analysis revealed no significant differences in standing long jump performance among females, whereas males exhibited significant differences throughout the study period (MD = 4.87 cm). Significant differences were observed in both groups when stratified by year group, with junior secondary students (MD = 4.841 cm) showing markedly superior results to senior secondary students (MD = 3.114 cm). When grouped by intervention duration, the < 18-week group (MD = 4.771 cm) demonstrated significantly better outcomes than the ≥18-week group (MD = 3.780 cm).

**Table 6:** Meta-regression of different moderating variables on standing long jump performance in secondary school students.

**Regulating variable**	**Coefficient of regression**	**Standard error**	***T*-value**	***P* > |*t*|**	**95% CI**
Gender	−3.213031	3.61721	−0.89	0.398	−11.39573, 4.969667
Year	−1.442842	4.754725	−0.30	0.768	−12.19878, 9.313093
Intervention cycle	−0.6307254	4.50467	−0.14	0.892	−10.821, 9.559546

**Table 7:** Subgroup analysis of the effects of different modifying variables on standing long jump performance among secondary school students.

**Regulating variable**	**Stratified subgroup**	**Number of studies included**	**MD (95% CI)**	***Z*-value**	***P*-value**
Gender	Male	8	4.870 cm (2.534, 7.207)	4.08	0.007
	Female	5	3.512 cm (−0.470, 7.493)	1.73	0.084
Year	Junior high school student	9	4.841 (2.902, 6.780)	4.89	0.000
	High school student	4	3.114 cm (0.113, 6.115)	2.03	0.042
Intervention cycle	< 18 weeks	7	4.771 cm (2.874, 6.668)	4.93	0.000
	≥18 weeks	6	3.780 cm (0.359, 7.201)	2.17	0.030

##### 3.3.3.4 Sensitivity test

**Figure 13:**
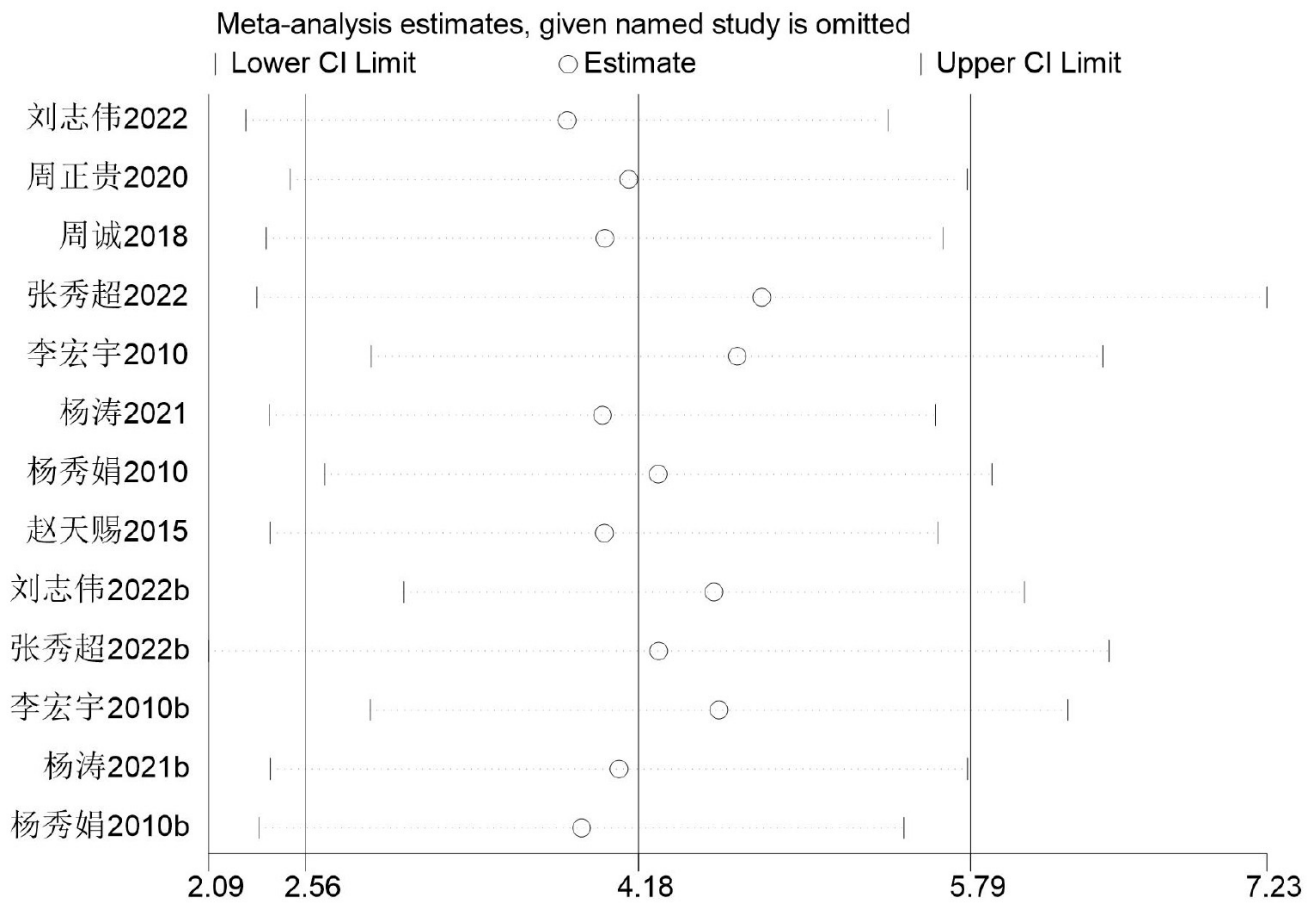
Sensitivity of standing long jump performance in secondary school students to basketball training.

Firstly, switching between random-effects and fixed-effects models and re-conducting statistical analyses revealed no significant alterations in all statistical outcomes. Secondly, assessing the influence of each study by sequentially excluding one study demonstrated that the pooled effect size MD (2.56 cm, 5.79 cm) and *I*^2^ ranged between 67 and 74%, with *P* < 0.00001 (Figure 13).

#### 3.3.4 The effects of basketball on muscle strength in Chinese middle school students

##### 3.3.4.1 Integrity check

**Figure 14:**
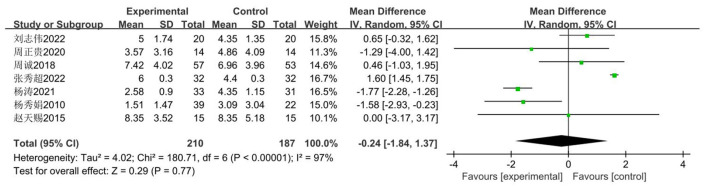
Forest plot of pull-up performance among secondary school students participating in basketball.

The pull-up is commonly used as an indicator for assessing the physical fitness of male middle school students in China, primarily evaluating their muscular strength. The impact of basketball training on pull-up performance among secondary school students was examined across seven studies (34–41), involving 397 participants: 210 in the experimental group and 187 in the control group. Heterogeneity testing revealed substantial heterogeneity (*I*^2^ = 97, *P* < 0.00001). Consequently, a random-effects model was employed. As depicted in Figure 14, the results intersect with the zero line, indicating no statistically significant difference (*P* > 0.05).

##### 3.3.4.2 Meta-regression and moderator subgroup analysis

Given that the heterogeneity of the pooled effect exceeds 50%, analysis will be conducted based on the included studies. Meta-regression analysis will be performed with grade level and intervention duration as moderator variables. As shown in Table 8 and the subgroup analysis in Table 9, no significant differences were observed (*P* = 0.77 > 0.05). Consequently, it can be concluded that basketball training exerts a negligible impact on middle school students' pull-up performance, with no statistically significant differences identified.

**Table 8:** Meta-regression of different moderating variables on secondary school pupils' pull-up performance.

**Regulating variable**	**Coefficient of regression**	**Standard error**	***T*-value**	***P* > |*t*|**	**95% CI**
Gender	1.016873	1.525764	0.67	0.542	−3.219327, 5.253072
Year	−1.13652	1.306303	−0.87	0.433	−4.763398, 2.490357

**Table 9:** Subgroup analysis of different modifying variables on secondary school students' pull-up performance.

**Regulating variable**	**Stratified subgroup**	**Number of studies included**	**MD (95% CI)**	***Z*-value**	***P*-value**
Year	Junior high school student	5	−0.409 t (−2.342, 1.523)	0.42	0.678
	High school student	2	0.377 t (−0.973, 1.726)	0.55	0.585
Intervention cycle	< 18 weeks	4	0.132 t (−2.038, 2.303)	0.12	0.905
	≥18 weeks	3	−0.736 t (−2.177, 0.705)	1.00	0.317

##### 3.3.4.3 Sensitivity test

**Figure 15:**
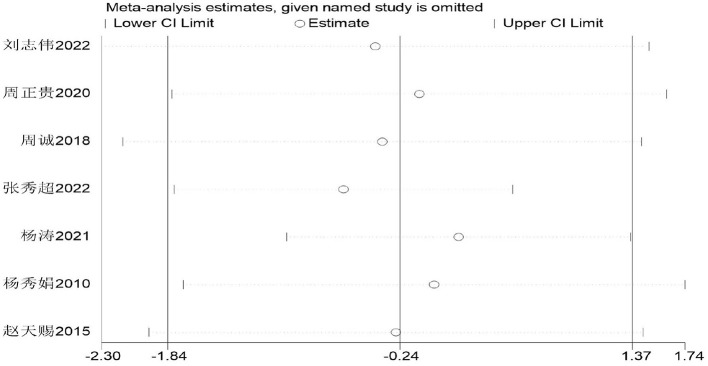
Sensitivity of basketball training to pull-up performance in secondary school students.

Firstly, switching between random-effects and fixed-effects models and re-conducting statistical analyses revealed no significant alterations in all statistical outcomes. Secondly, assessing the influence of each study by sequentially excluding one study demonstrated a pooled effect size of MD (−1.84 t, 1.37 t), with *I*^2^ ranging between 80 and 97% (Figure 15).

##### 3.3.4.4 Integrity check

**Figure 16:**
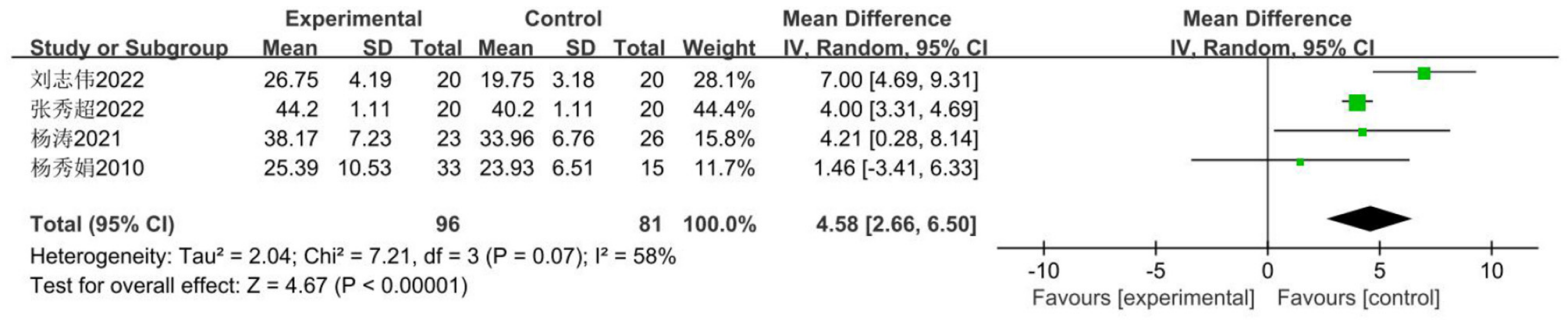
Forest plot illustrating the impact of basketball participation on secondary school pupils' sit-up performance.

For Chinese female middle school students, the standard test for muscle strength is typically sit-ups. The impact of basketball participation on secondary school pupils' sit-up performance was examined across four studies (34–36, 41), involving 177 secondary school students: 96 in the experimental group and 81 in the control group. The studies exhibited high heterogeneity (*I*^2^= 58%), necessitating a random-effects model for effect testing. Results (Figure 16) demonstrate a significant improvement in the experimental group compared to the control group [MD = 4.58 t, 95% CI (2.66, 6.50), *P* < 0.00001].

##### 3.3.4.5 Meta-regression and moderator subgroup analysis

Given that the heterogeneity of the pooled effect exceeds 50%, analysis will be conducted based on the included studies. Meta-regression analysis will be performed with intervention duration as the moderator variable. As shown in Table 10 and the subgroup analysis in Table 11, basketball activities lasting less than 18 weeks significantly improved students' sit-up performance (MD = 5.003), whereas no significant difference was observed for durations exceeding 18 weeks.

**Table 10:** Meta-regression of different moderating variables on secondary school students' sit-up performance.

**Regulating variable**	**Coefficient of regression**	**Standard error**	***T*-value**	***P* > |*t*|**	**95% CI**
Intervention cycle	−3.533088	3.058709	−1.16	0.367	−16.69365, 9.627474

**Table 11:** Subgroup analysis of different moderating variables on secondary school students' sit-up performance.

**Regulating variable**	**Stratified subgroup**	**Number of studies included**	**MD (95% CI)**	***Z*-value**	***P*-value**
Intervention cycle	< 18 weeks	3	5.003 t (2.888, 7.119)	4.64	0.000
	≥18 weeks	1	1.460 t (−3.415, 6.335)	0.59	0.557

##### 3.3.4.6 Sensitivity analysis

**Figure 17:**
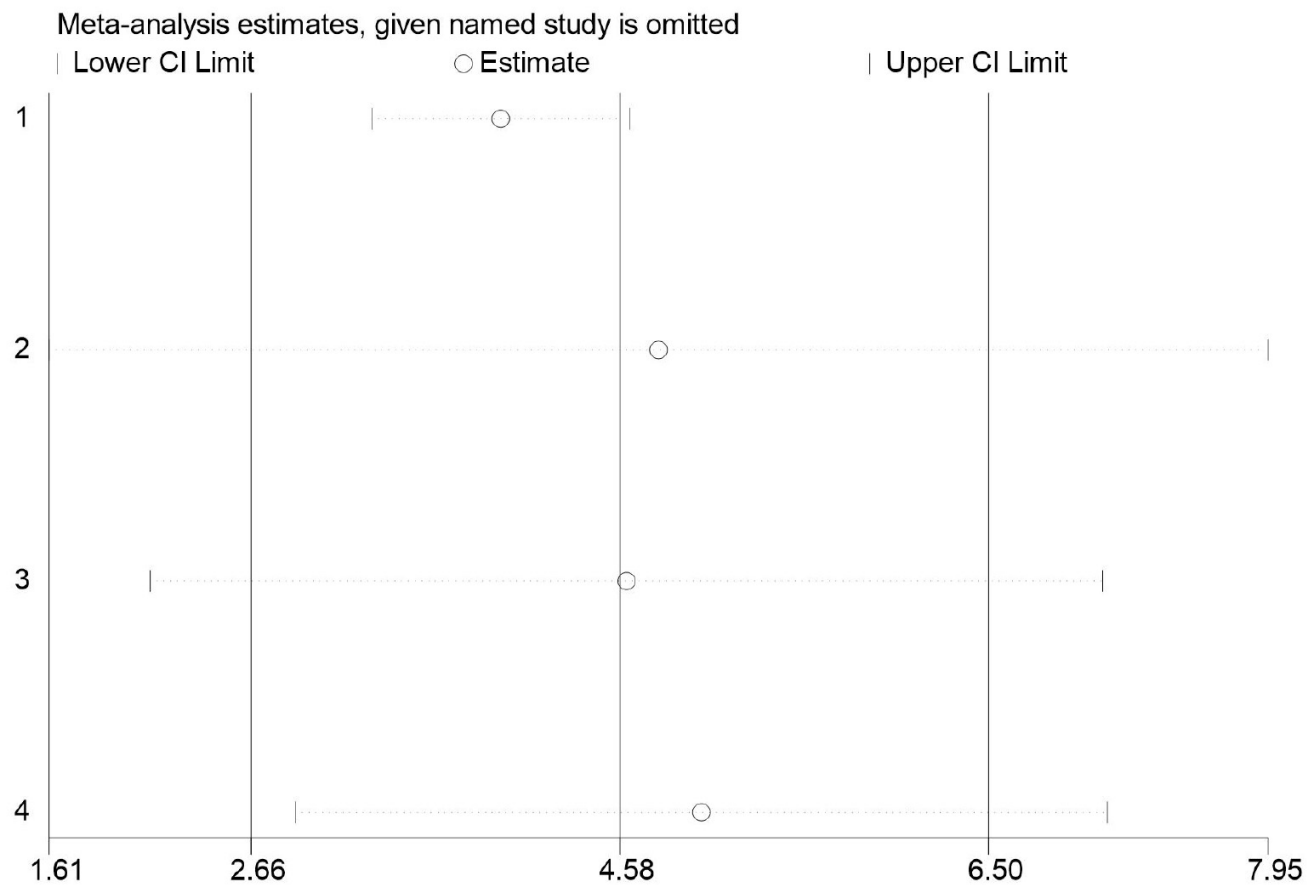
Sensitivity analysis of basketball training on secondary school students' sit-up performance.

Firstly, switching between random-effects and fixed-effects models and re-conducting statistical analyses revealed no significant alterations in all statistical outcomes. Secondly, assessing the influence of each study by sequentially excluding one study demonstrated that the pooled effect size remained unchanged MD (2.66 t, 6.50 t) (Figure 17).

#### 3.3.5 The impact of basketball on the endurance fitness of Chinese middle school students

##### 3.3.5.1 Integrity check

**Figure 18:**
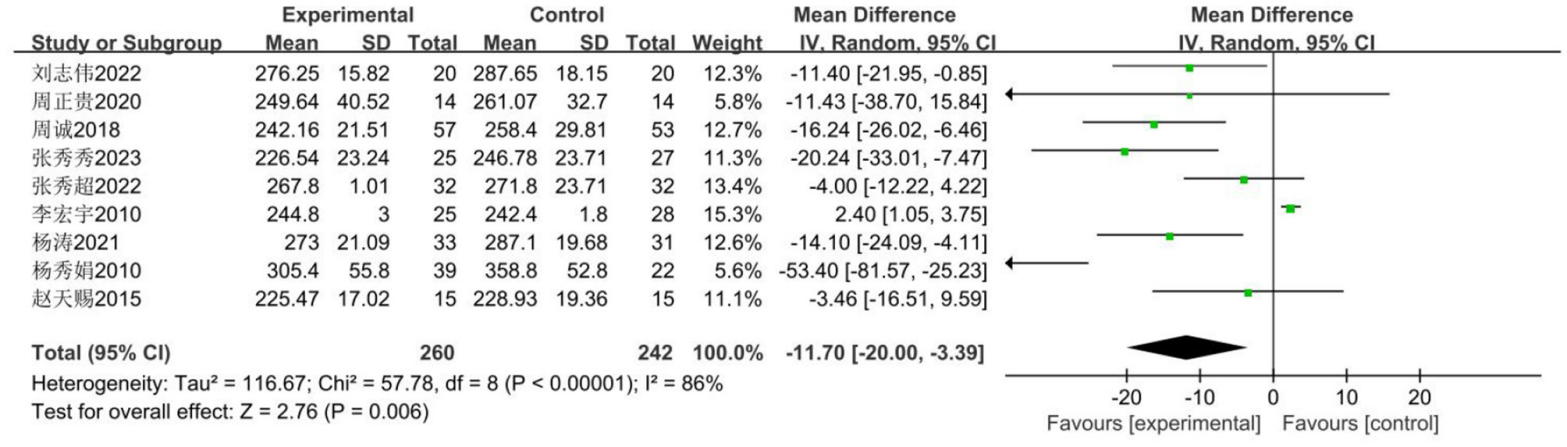
Forest plot illustrating the impact of basketball participation on secondary school pupils' 1,000 m performance.

Among male middle school students in China, the 1,000-m run is commonly used as an indicator for evaluating endurance fitness. Nine studies (33–41), involving 502 secondary school students: 260 in the experimental group and 242 in the control group. The analysis revealed high heterogeneity (*I*^2^ = 86%), necessitating a random-effects model for effect testing. Results (Figure 18) demonstrated a statistically significant improvement in the experimental group compared to the control group [MD = −11.70 s, 95% CI (−20.00, −3.39), *P* = 0.006 < 0.05].

##### 3.3.5.2 Publication bias assessment

**Figure 19:**
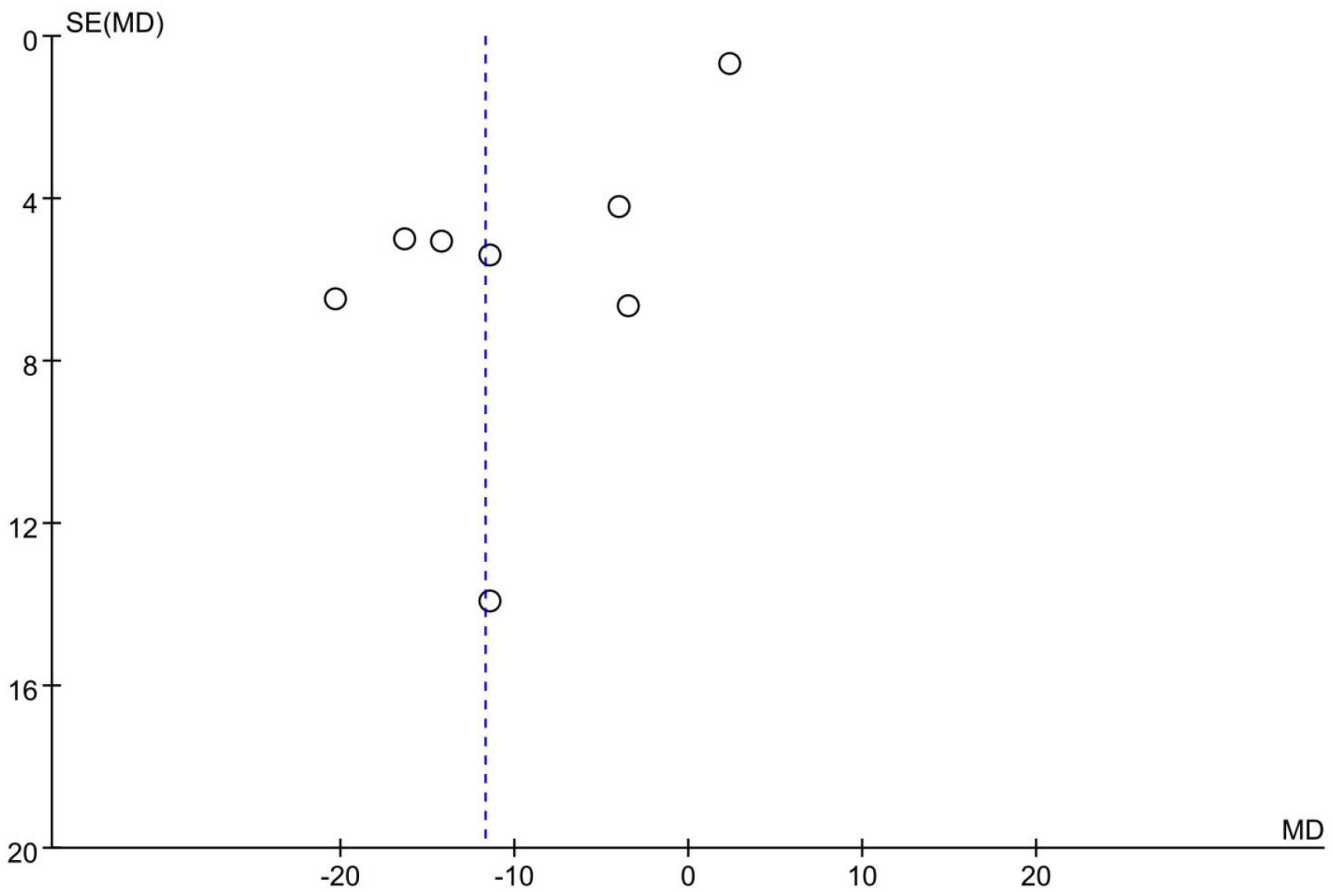
Funnel chart of the impact of basketball training on secondary school students' 1,000 m performance.

As illustrated in Figure 19, the published articles are evenly distributed on both sides of the mean effect size, indicating no apparent publication bias. Further analysis via Begg's Test reveals *P* = 0.466 > 0.05, thus confirming the absence of significant publication bias.

##### 3.3.5.3 Meta-regression and moderator subgroup analysis

Given that the pooled effect heterogeneity exceeded 50%, analysis will be conducted based on the included studies. Meta-regression analysis will be performed with grade level and intervention cycle as moderator variables. As per Table 12 and the subgroup analysis in Table 13, significant differences were observed between the control and experimental groups in the junior secondary cohort, with the experimental group demonstrating shorter completion times (MD = −14.090 s). However, no significant difference was found between the experimental and control groups in the senior secondary cohort (*P* = 0.170 > 0.05). Regarding intervention duration, significant differences were observed in the < 18-week group, where the experimental group completed tasks faster than the control group (MD = −10.156 s), whereas no significant difference was found in the ≥18-week group (*P* = 0.086 > 0.05).

**Table 12:** Meta-regression of the effects of different moderating variables on 1,000 m performance.

**Regulating variable**	**Coefficient of regression**	**Standard error**	***T*-value**	***P* > |*t*|**	**95% CI**
Year	8.254554	10.21637	0.81	0.450	−16.74401, 33.25312
Intervention cycle	−6.193772	10.45025	−0.59	0.575	−31.7646, 19.37706

**Table 13:** Subgroup analysis of the effect of different modifying variables on 1,000 m performance.

**Regulating variable**	**Stratified subgroup**	**Number of studies included**	**MD (95% CI)**	***Z*-value**	***P*-value**
Year	Junior high school student	5	−14.090 s (−24.446, −3.734)	2.67	0.008
	High school student	4	−8.748 s (−21.233, 3.737)	1.37	0.170
Intervention cycle	< 18 weeks	5	−10.156 s (−16.082, −4.231)	3.36	0.001
	≥18 weeks	4	−16.114 s (−34.486, 2.259)	1.72	0.086

##### 3.3.5.4 Sensitivity analysis

**Figure 20:**
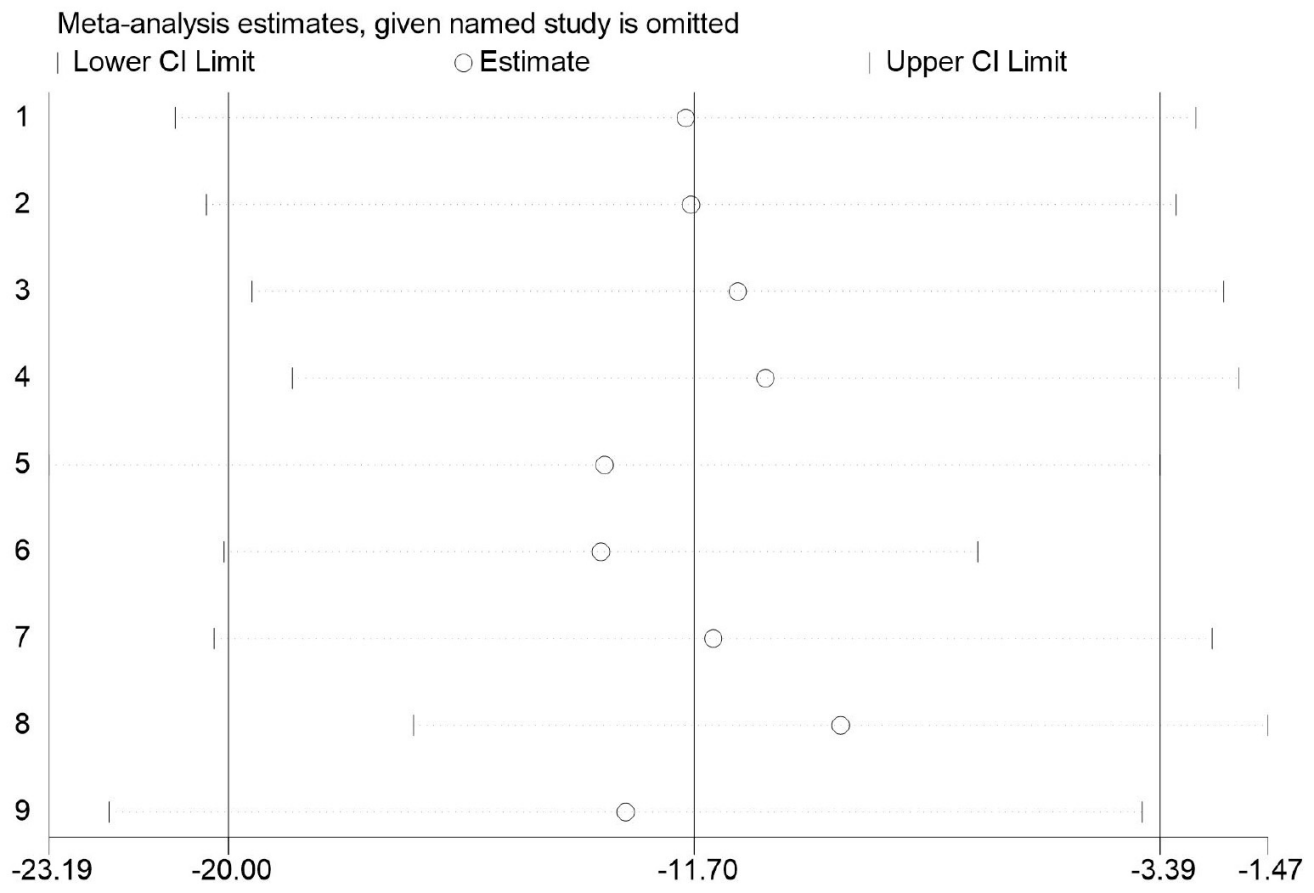
Sensitivity of basketball training to secondary school students' 1,000 m performance.

Firstly, switching between random-effects and fixed-effects models and re-conducting statistical analyses revealed no significant alterations in all statistical outcomes. Secondly, assessing the influence of each study by sequentially excluding one study demonstrated a pooled effect size of MD (−20.00, −3.39), with *I*^2^ ranging between 56 and 88% (Figure 20).

##### 3.3.5.5 Integrity check

**Figure 21:**
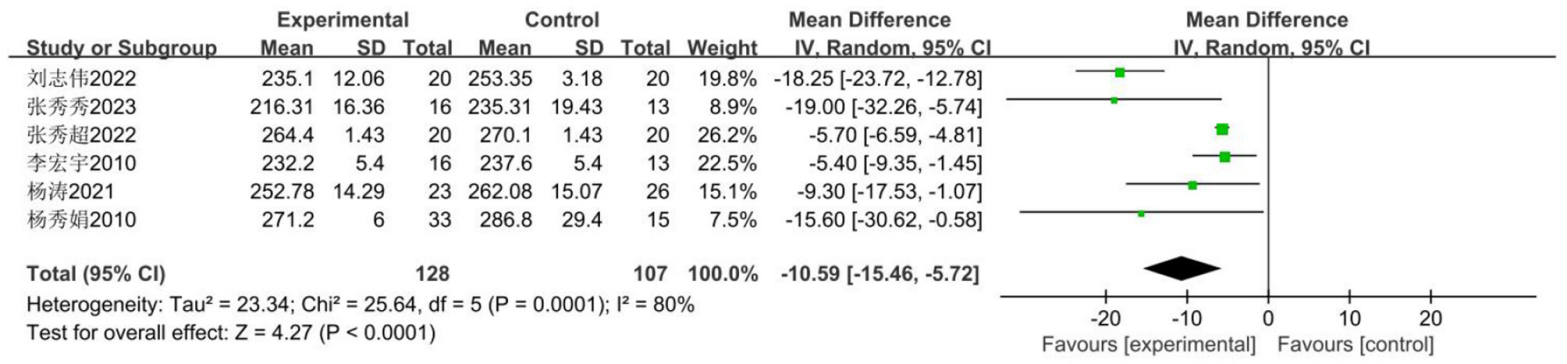
Forest plot illustrating the impact of basketball participation on secondary school pupils' 800 m performance.

For Chinese female middle school students, the 800-m run serves as the primary assessment metric for endurance fitness. The impact of basketball training on secondary school pupils' sit-up performance included six studies (33–37, 41), involving 235 secondary school students: 128 in the experimental group and 107 in the control group. The analysis revealed high heterogeneity (*I*^2^ = 80%), necessitating a random-effects model for effect testing. Results (Figure 21) demonstrated a statistically significant improvement in the experimental group compared to the control group [MD = −10.59 s, 95% CI (−15.46, −5.72), *P* < 0.0001].

##### 3.3.5.6 Meta-regression and moderator subgroup analysis

Given that the pooled effect heterogeneity exceeded 50%, analysis will be conducted based on the included studies. Meta-regression analysis will be performed using grade level and intervention cycle as moderator variables. As per Table 14 and the subgroup analysis in Table 15, significant differences were observed between the control and experimental groups in the junior secondary cohort, with the experimental group demonstrating shorter completion times (MD = −11.589 s). However, no significant difference was found between the experimental and control groups in the senior secondary cohort (*P* = 0.107 > 0.05). Regarding intervention duration, significant differences were observed in the < 18-week group, with the experimental group demonstrating shorter completion times than the control group (MD = −12.278 s). However, no significant difference was found in the ≥18-week group (*P* = 0.072 > 0.05).

**Table 14:** Meta-regression of different moderating variables on secondary school pupils' 800 m performance.

**Regulating variable**	**Coefficient of regression**	**Standard error**	***T*-value**	***P* > |*t*|**	**95% CI**
Year	−0.5224525	7.957535	−0.07	0.952	−25.84688, 24.80198
Intervention cycle	3.786944	8.055137	0.47	0.670	−21.8481, 29.42198

**Table 15:** Subgroup analysis of different modifying variables on secondary school students' 800 m performance.

**Regulating variable**	**Stratified subgroup**	**Number of studies included**	**MD (95% CI)**	***Z*-value**	***P*-value**
Year	Junior high school student	4	−11.589 s (−19.385, −3.793)	2.91	0.004
	High school student	2	−10.667 s (−23.651, 2.318)	1.61	0.107
Intervention cycle	< 18 weeks	5	−12.278 s (−20.260, −4.296)	3.01	0.003
	≥18 weeks	4	−7.821 s (−16.327, 0.685)	1.80	0.072

##### 3.3.5.7 Sensitivity analysis

**Figure 22:**
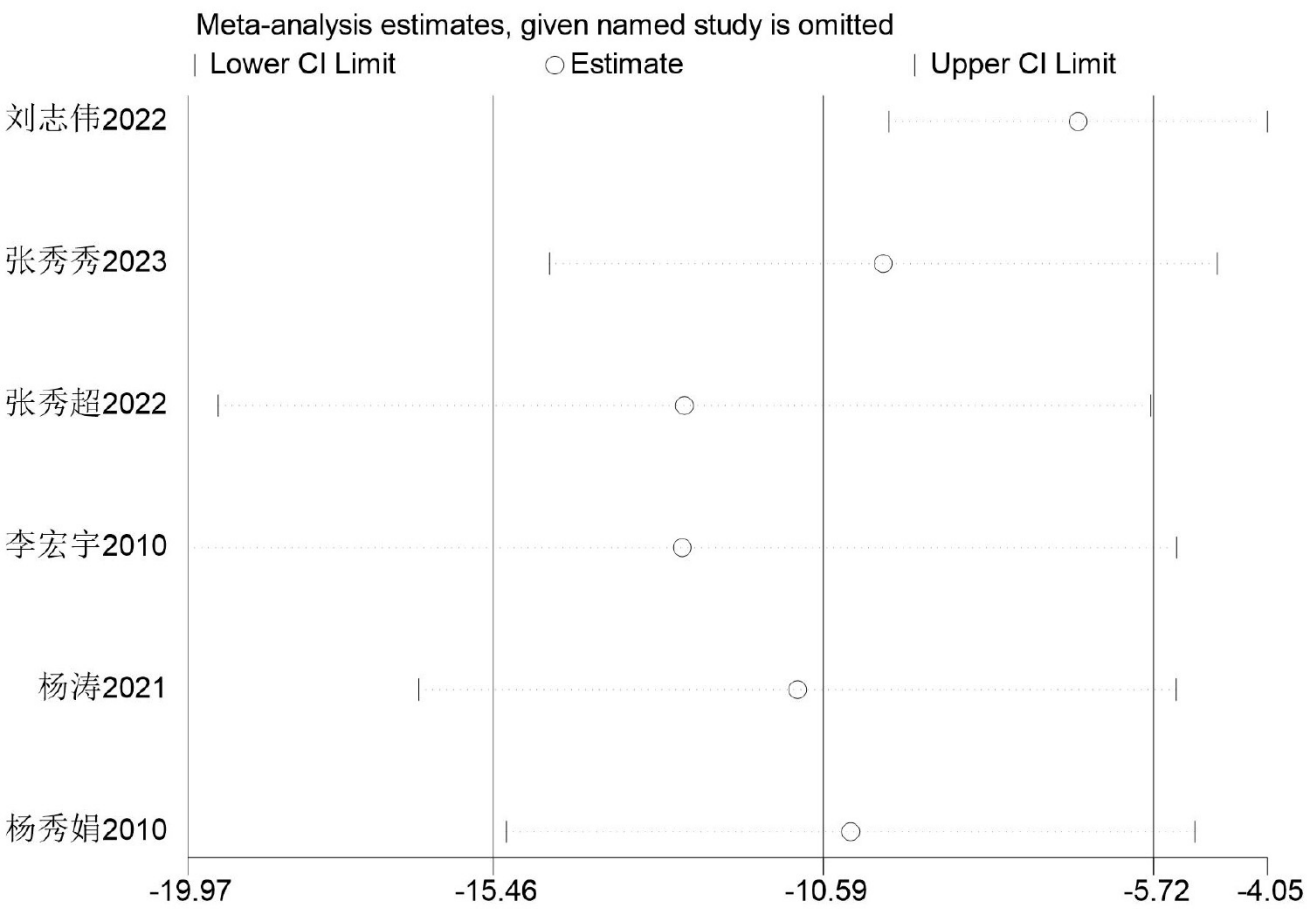
Sensitivity analysis of basketball training on secondary school students' 800 m performance.

Firstly, switching between random-effects and fixed-effects models and re-conducting statistical analyses revealed no significant alterations in all statistical outcomes. Secondly, assessing the influence of each study by sequentially excluding one study demonstrated that the pooled effect size MD (−15.46 s, −5.72 s) and *I*^2^ ranged between 36 and 84% (Figure 22).

## 4 Discussion

### 4.1 The impact of basketball on the physical health of Chinese middle school students

Currently, driven by health initiatives such as China's “Year of Weight Loss,” the physical fitness of adolescents has become a focal point of societal concern (42). Xiaofei R (2023) suggests that basketball has little impact on 50 m sprint and pull-up performance, but significantly influences sit-ups, sit-and-reach, 800, and 1,000 m outcomes (40). Huiju Z (2008) contends that basketball develops various fundamental skills among secondary students—such as running, jumping, and throwing—enhances physical fitness, and improves psychological regulation abilities (43). Zhang L (2016) asserts that basketball has certain benefits for the physical health of primary and secondary school students (44). This study systematically evaluates the impact of basketball on multiple key physical fitness indicators among secondary students. Basketball significantly improved middle school students' performance in the 50-m dash (MD = −0.41 s), sit-and-reach (MD = 2.22 cm), standing long jump (MD = 4.18 cm), sit-ups (MD = 5.003 reps), the 1,000-m run for males (MD = −11.70 s), and the 800-m run for females (MD = −10.59 s). Multiple scholars suggest these indicators can partially represent students' overall physical qualities (46, 47). As a comprehensive sport, basketball offers benefits for middle school students' speed, flexibility, explosive power, core strength, and cardiorespiratory endurance. Subgroup analyses by gender, age, and intervention duration revealed that females outperformed males in the 50 m sprint; junior high students outperformed senior high students; and those with < 18 weeks of intervention outperformed those with ≥18 weeks. Subgroup analysis of the sit-and-reach test results showed males outperformed females; the junior high experimental group outperformed the control group; and the < 18-week experimental group outperformed the control group. Subgroup analysis of basketball's effect on standing long jump performance showed: male experimental group outperformed the control group; junior high students outperformed senior high students; and the < 18-week group outperformed the ≥18-week group. Subgroup analysis of basketball's effect on sit-up performance indicated the < 18-week experimental group significantly outperformed the control group. Subgroup analysis of basketball's impact on middle school students' 1,000 m performance revealed that the junior high experimental group outperformed the control group; the < 18 weeks experimental group outperformed the control group. Subgroup analysis of basketball's impact on middle school students' 800 m performance showed that the junior high experimental group outperformed the control group; the < 18 weeks experimental group outperformed the control group. During subgroup analysis, it was found that gender, grade level, and intervention cycle primarily influenced the results. However, during the final presentation of findings, significant heterogeneity was observed in certain indicators (potentially stemming from regional differences, intervention intensity, and duration). Due to missing data in some studies, more detailed categorization was not feasible. Furthermore, the overall quality of the included original literature was relatively low. Future research is encouraged to pursue more refined categorization and precise exploration. Physical education teachers and health professionals may utilize basketball as a means to enhance students' physical fitness. However, during basketball instruction, attention should be paid to individual differences and differentiated teaching: research indicates that heterogeneity correlates with factors such as grade level, gender, and intervention intensity, suggesting that a uniform basketball training program may yield limited effectiveness. Research indicates that pull-up performance does not significantly impact overall physical fitness. Therefore, incorporating specific upper-body strength development exercises into basketball training sessions aims to achieve comprehensive physical development among middle school students. Teachers should design training plans of varying intensity and focus based on students' age, gender, physical fitness foundation, and interest level. While promoting basketball participation to enhance physical health among secondary students, attention must be paid to individual differences and injury prevention (48). International youth sports guidelines consistently emphasize that premature specialization in a single sport may increase injury risks and lead to psychological burnout. Therefore, educators should highlight the importance of diverse physical activities alongside basketball promotion, integrating injury prevention knowledge into instruction. Basketball should serve as a tool to promote healthy lifestyles among secondary school students, not as the sole means to that end.

### 4.2 Conclusions

The purpose of this study is to provide evidence-based support for whether basketball contributes to the physical fitness of Chinese middle school students. Through a review of lower-quality literature, it was found that basketball positively impacts the physical fitness of middle school students. Specifically, improvements were observed in various physical fitness components as measured by specific indicators: the 50 m sprint time, representing speed fitness, showed improvement; the sit-and-reach test, representing flexibility fitness, also improved; and the standing long jump distance, representing lower-body strength fitness, also improved. There was no significant improvement in pull-up performance, which represents upper-body strength. Sit-up performance, representing overall strength, also improved. Endurance performance, as measured by 800 and 1,000 m run times, also showed improvement. In summary, basketball can enhance middle school students' speed, endurance, flexibility, and strength, thereby comprehensively improving their physical fitness. However, it did not significantly improve pull-up performance.

### 4.3 Limitations

This study primarily explored the impact of basketball on the physical fitness of secondary school students. Despite strict adherence to the PRISMA guidelines and PICOST criteria, certain limitations and shortcomings remain. All included studies were conducted within China, lacking geographical diversity, and the extent to which their findings can be broadly generalized remains uncertain, during the research process, the varying intervention intensities and duration across some literature may pose challenges to the study's generalizability. The search and inclusion process was limited to Chinese and English literature, excluding other languages and failing to broaden the scope further. This raises the possibility that relevant studies may have been overlooked. Although publication bias checks and sensitivity analyses indicated no significant bias in the results, factors such as insufficient sample sizes in the included studies suggest a potential risk of bias in the research.

## Data Availability

The datasets presented in this study can be found in online repositories. The names of the repository/repositories and accession number(s) can be found below: All data in this paper are presented in scienceDB at 10.57760/sciencedb.23826.
