# Supplementary material for: The impact of basketball on the physical health of Chinese middle school students aged 12–18: a systematic review and meta-analysis
Source: Front Public Health. 2025 Dec 19;13:1692668. doi: 10.3389/fpubh.2025.1692668 (PMC12757973; doi:10.3389/fpubh.2025.1692668)
Supplement: Supplementary file 1 [file Data_Sheet_1.pdf]

目录

1. Unit Supplement 2

2. Research Registration 2

3. Search Query 2

4. Search Flowchart 4

5. Reference Verification: 5

6. Cochrane Evaluation Criteria 7

7. Bias Risk Assessment 10

8. PRISMA 2020 Checklist 12

1.Unit Supplement

S：second

T：times

M：Metric

Cm：centimeter

2.Research Registration

To prevent duplicate research, this study was prospectively registered on PROSPERO CRD420251022157, ensuring the research methodology fully aligns with the registered protocol.

3.Search Query

((("Basketball"[Mesh]) OR ((((Basketball) OR (Basketballs)) OR (Netball)) OR (Netballs))) AND (((Middle school student) OR (middle school students)) OR (high school students))) AND (((((((((physical health) OR (physical health status)) OR (50-metre sprint)) OR (sit-and-reach)) OR (standing long jump)) OR (pull-ups)) OR (one-minute sit-ups)) OR (1000-metre run)) OR (800-metre run))

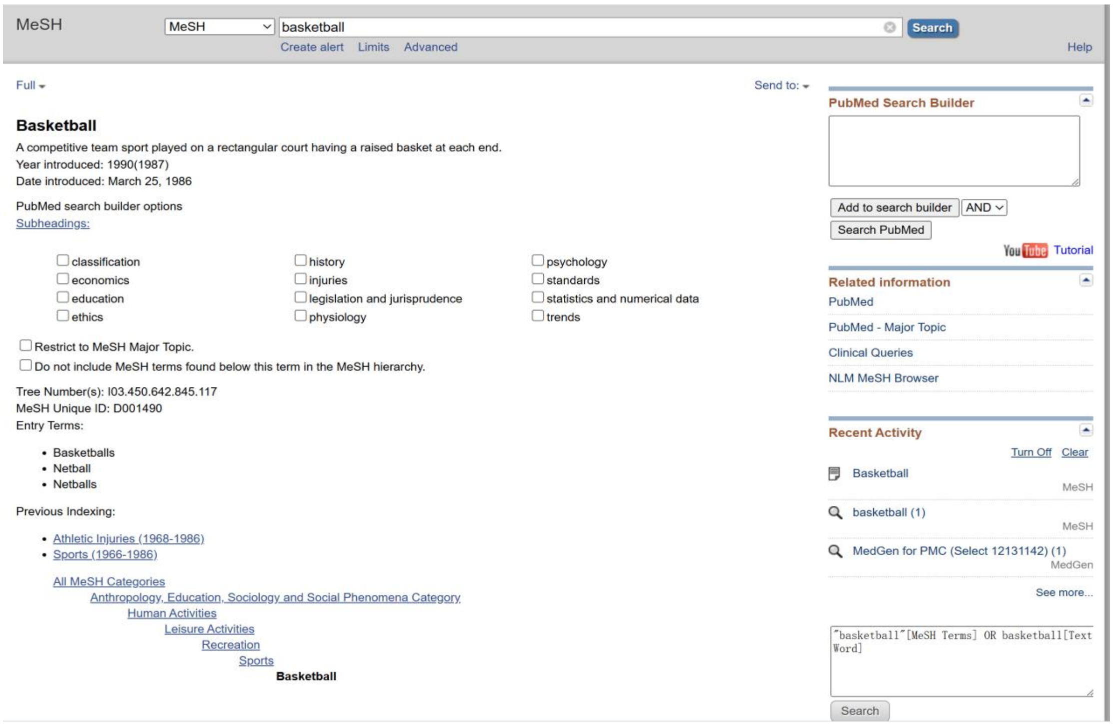
MeSH
MeSH
basketball
Search
Create alert Limits Advanced
Help
Full
Send to:
Basketball
A competitive team sport played on a rectangular court having a raised basket at each end.
Year introduced: 1990(1987)
Date introduced: March 25, 1986
PubMed search builder options
Subheadings:
classification
economics
education
ethics
history
injuries
legislation and jurisprudence
physiology
psychology
standards
statistics and numerical data
trends
Restrict to MeSH Major Topic.
Do not include MeSH terms found below this term in the MeSH hierarchy.
Tree Number(s): I03.450.642.845.117
MeSH Unique ID: D001490
Entry Terms:
Basketballs
Netball
Netballs
Previous Indexing:
Athletic Injuries (1968-1986)
Sports (1966-1986)
All MeSH Categories
Anthropology, Education, Sociology and Social Phenomena Category
Human Activities
Leisure Activities
Recreation
Sports
Basketball
PubMed Search Builder
Add to search builder
AND
Search PubMed
Tutorial
Related information
PubMed
PubMed - Major Topic
Clinical Queries
NLM MeSH Browser
Recent Activity
Turn Off
Clear
Basketball
MeSH
basketball (1)
MeSH
MedGen for PMC (Select 12131142) (1)
MedGen
See more...
"basketball"[MeSH Terms] OR basketball[Text Word]
Search

4.Search Flowchart

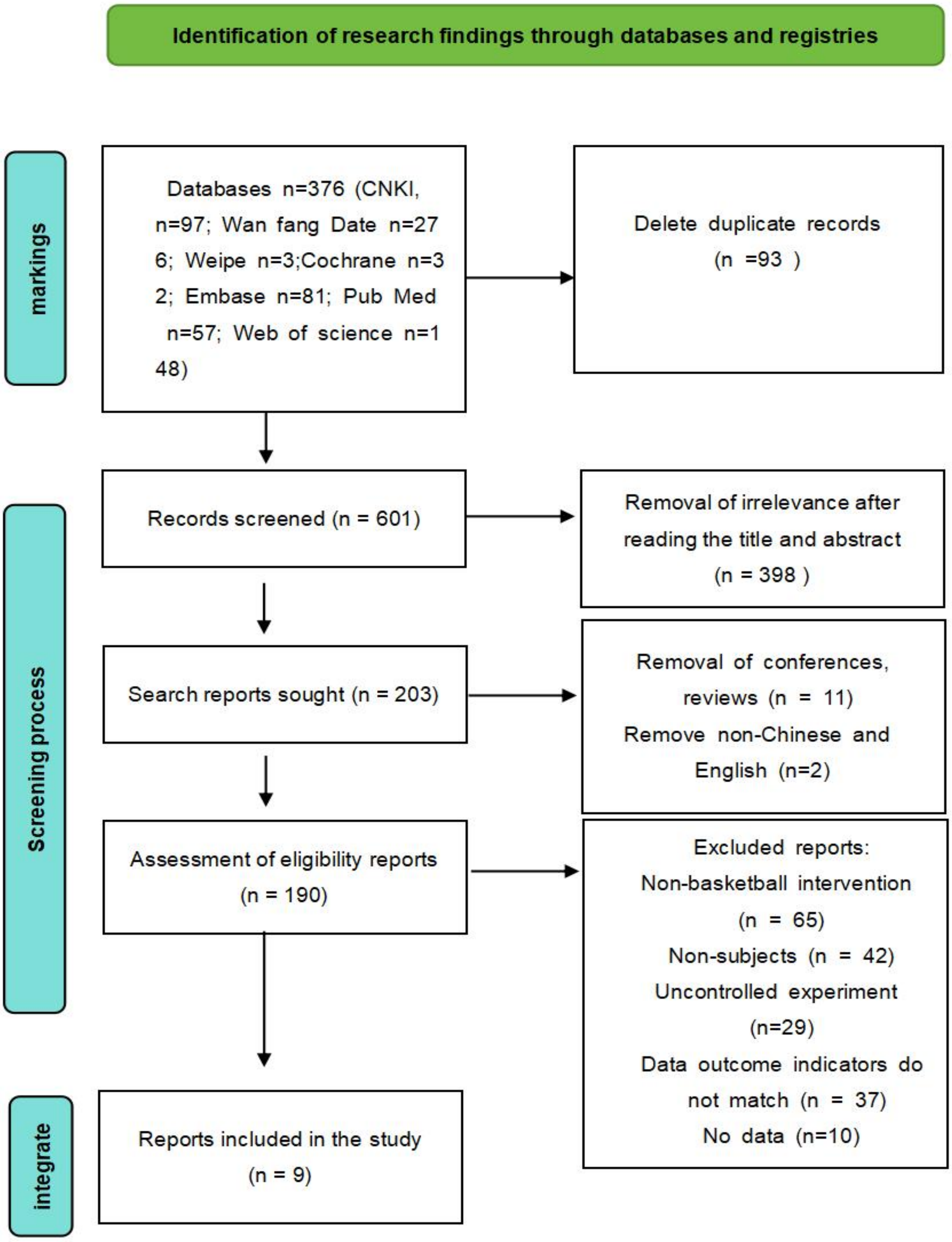

5.Reference Verification:

Literature10: Yang Xiujuan. Experimental Study on the Fitness Effects of Ball Sports Exercise Programmes for Secondary School Students [Doctoral Thesis]. Xi'an Sport University, 2010.

Search Results Page：

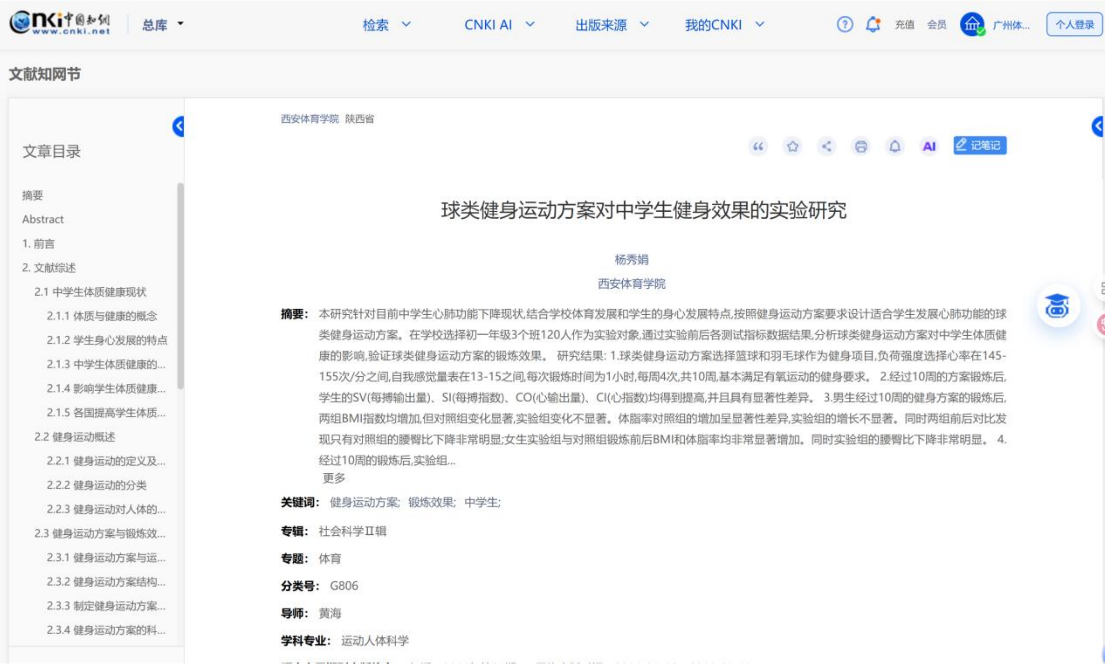

Literature link:

https://kns.cnki.net/kcms2/article/abstract?v=BP09Z8g3bnKAIG_spzzG422F71X5XW4isBEFxrucyHkoFXHhDb-SWk86FqCW2vR0rgjlKdw2_17T-FzOmfDltiTNkDobMyXMKMaMCB1gmiP6tWpkiNq_wOmTa9fGQLGv44-305LpAgVE1QJJuUn3U6cskyZ3BAPm_qva8cmdS3zrov_ikOvLwg==&uniplatform=NZKPT&language=CHS

Literature14: Zhou Cheng. Experimental Research on the Application of Sports Education Curriculum Models in Secondary School Physical Education Teaching: Taking Basketball as an Example [D]. Hunan University of Technology, 2018.

Search Results Page：

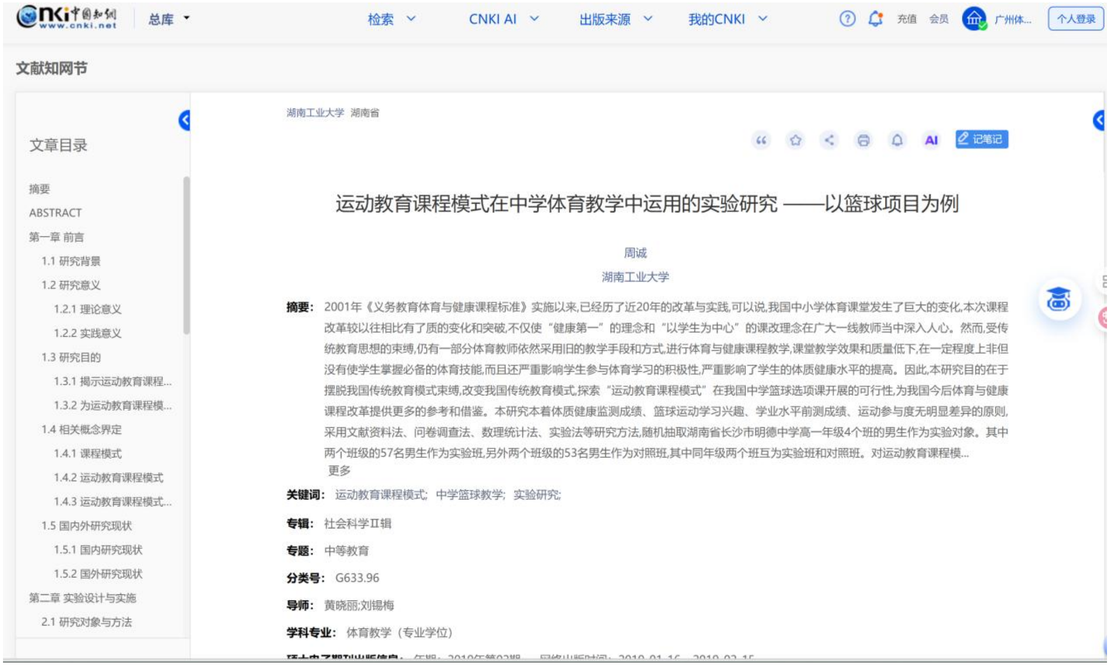

Literature link:

https://kns.cnki.net/kcms2/article/abstract?v=BP09Z8g3bnJsGP0GsujTCLvube0Se7jVM_0A7WKHopdUgE4ArsqnFYaPpsg_ImCUk-6CLvltWPpMGl0xFkQg_FXDqQV0U2BPyWmDhRFB5WfxifPaqXEE6JB_bP-WGu_hXhR1Uqpv2ZH59ei2MqYhjDusms61JKjj2nmN98JR4nnalkfJTZHkarxNQW4C-arI&uniplatform=NZKPT&language=CHS

6.Cochrane Evaluation Criteria

Random sequence generation<br>Inappropriate methods for generating random sequences leading to selection bias (intervention allocation bias)	
Criteria for Assessing<br>Low Bias Risk	Researchers described random methods during the sequence generation process, such as:<br>Random number tables, computer-generated random numbers, coin tossing, shuffling or envelopes, dice rolling, lottery draw,minimisation method
Criteria for Assessing<br>High Bias Risk	Researchers described non-random methods employed during sequence generation. Typically, such descriptions encompass systematic, non-random approaches including: allocation sequences based on whether birthdays were odd or even; allocation determined by the judgement of the intervention implementer; allocation according to subject preference; allocation based on baseline test results or a series of examination outcomes; and allocation according to the efficacy of the intervention.
Criteria for Assessing Uncertainty in Bias Risk	The information generated by the sequence is unclear, making it difficult to determine whether it is 'low risk' or 'high risk'.
Allocation Concealment<br>Selection bias arising from inadequate concealment of the follow-up allocation scheme (bias in the allocation of interventions)	
Criteria for Assessing<br>Low Bias Risk	Neither the subjects nor the researchers recruiting them can foresee the allocation outcome, as the randomisation scheme is concealed by employing the following methods or equivalent approaches: Grouping is conducted by a third party, Interventions are administered by a third party, etc.
Criteria for Assessing<br>High Bias Risk	Researchers conducting or recruiting subjects may anticipate allocation outcomes, leading to selection bias, as in the following allocation methods: where the experiment designer and implementer are the same individual, or other methods where concealment is demonstrably impossible.

Criteria for Assessing Uncertainty in Bias Risk	Insufficient information exists to determine whether the risk is "low" or "high". Typically, this arises when the method is not described, or is described inadequately, preventing a clear assessment.
Implementation of blinding for subjects and trial personnel<br>Implementation bias arising from the allocation of interventions in the study being known to both participants and trial personnel.	
Criteria for Assessing Low Bias Risk	The presence of any one of the following: no blinding or inadequate blinding, but the systematic reviewer judges that outcomes would not be affected by the lack of blinding; blinding implemented for both participants and primary investigators, and this blinding is not compromised.
Criteria for Assessing High Bias Risk	Any one of the following applies: Blinding was not employed or was inadequate, potentially affecting outcome assessment or measurement; Blinding was implemented for subjects and key investigators, but this blinding could be compromised.
Criteria for Assessing Uncertainty in Bias Risk	The presence of any one of the following: insufficient information to determine "yes" or "no"; the outcome measure was not reported in the study.
Implementing a blinded approach for outcome assessors<br>Implementation bias arising from outcome assessors' knowledge of intervention group assignments	
Criteria for Assessing Low Bias Risk	Any one of the following applies: Blinding was not implemented, but the systematic reviewer judged that outcome measurement would not be affected by the lack of blinding; Blinding was implemented for outcome measurers, and this blinding was not compromised.
Criteria for Assessing High Bias Risk	Any one of the following applies: Blinding was not employed or was inadequate, potentially influencing outcome assessment or measurement; Blinding was implemented for outcome assessors, but this blinding could be compromised.
Criteria for Assessing Uncertainty in Bias Risk	The presence of any one of the following: insufficient information to determine "yes" or "no"; the outcome measure was not reported in the study.
The results data is incomplete.<br>Follow-up bias arising from the quantity, variety and processing of incomplete outcome data	

Criteria for Assessing Low Bias Risk	The following conditions are met: There is no missing data, or missing data does not affect the analysis of results (e.g., missing values in survival analysis). The number and reasons for missing data are similar across groups. The effect size of the missing data (mean difference or standardised mean difference) is insufficient to significantly impact the observed effect size. Appropriate methods have been employed to handle the missing data.
Criteria for Assessing High Bias Risk	The presence of any one of the following: imbalance in the number and reasons for missing data between groups; the effect size of the missing data (mean difference or standardised mean difference) is insufficient to significantly impact the observed effect size; inappropriate methods for handling missing data.
Criteria for Assessing Uncertainty in Bias Risk	The following applies: Incomplete information makes it difficult to determine whether the data is complete (e.g., missing numbers or reasons not reported), and the study does not address issues of completeness.
Selective reporting<br>Reporting bias arising from selective reporting of results	
Criteria for Assessing Low Bias Risk	The following criteria apply: either a protocol exists and the systematic review reports all pre-specified outcome measures (primary and secondary) as intended; or no protocol exists, yet the published review reports all anticipated outcomes (including pre-specified ones), including those not explicitly defined (though compelling textual justification is less common).
Criteria for Assessing High Bias Risk	The presence of any one of the following: Failure to report all pre-specified primary outcome measures; One or more primary outcome measures reported using pre-unspecified measurements, data analysis methods, or data subsets (e.g., subscales); One or more primary outcome measures reported that were not pre-specified; Incomplete reporting of one or more outcome measures of interest to the systematic review, rendering them ineligible for inclusion in the meta-analysis; Failure to report important outcome measures.
Criteria for Assessing Uncertainty in Bias Risk	Incomplete information makes it difficult to determine whether there is a risk of selective reporting of results. It is likely that the majority of studies would be categorised as such.
Other biases<br>Bias not covered elsewhere in this form	
Criteria for Assessing Low Bias Risk	The study had no other sources of bias.

Criteria for Assessing High Bias Risk	At least one significant risk of bias exists. For example: the study has potential bias related to its particular research design; allegations of falsification have been made; and several other issues are present.
Criteria for Assessing Uncertainty in Bias Risk	There may be a risk of bias, or there may be insufficient information to determine whether a significant risk of bias exists, with no sufficient justification or evidence to suggest that this issue could lead to bias.

7.Bias Risk Assessment

Rank	Author	Year	Random sequence generation	Allocation concealment	Blinding of Participants and personnel	Blinding of outcome assessment	Incomplete outcome data	Selective reporting	Other bias
1	Liu Z W	2022	Allocation of participants to intervention groups in the study	The study explicitly stated that group concealment was not implemented.	No explicit reporting on whether subject blinding was implemented	The outcome measurement will not be affected.	A well-defined research proposal	Outcome data were complete.	The study had no other sources of bias.
2	Zhou Z G	2020	Intergroup allocation in the study was not randomized.	It is not specified whether grouping is hidden.	No blinding was performed in the study.	The outcome measurement will not be affected.	A well-defined research proposal	Outcome data were complete.	The study had no other sources of bias.
3	Zhou C	2018	Intergroup allocation in the study was not randomized.	Group concealment was explicitly implemented.	No explicit reporting on whether subject blinding was implemented	The outcome measurement will not be affected.	A well-defined research proposal	Outcome data were complete.	The study had no other sources of bias
4	Zhang X X	2023	Intergroup allocation in the study was not randomize	It is not specified whether grouping is hidden.	Implementation of blinding for both experimenters and subjects	The outcome measurement will not be affected.	A well-defined research proposal	Outcome data were complete.	The study had no other sources of bias

			d.						
5	Zhang X C	2022	Intergroup allocation in the study was not randomize d.	It is not specified whether grouping is hidden.	Implementati on of blinding for both experimenter s and subjects	The outcome measuremen t will not be affected.	A well-defined research proposal	Outcome data were complete.	The study had no other sources of bias
6	Li H Y	2010	Intergroup allocation in the study was not randomize d.	Group concealment was explicitly implemented.	No explicit reporting on whether subject blinding was implemented	The outcome measuremen t will not be affected.	A well-defined research proposal	Outcome data were complete.	The study results may be subject to certain biases due to characteristi cs of the research subjects.
7	Yang T	2021	Intergroup allocation in the study was not randomize d.	The study explicitly stated that group concealment was not implemented.	No blinding was performed in the study.	The outcome measuremen t will not be affected.	A well-defined research proposal	Outcome data were complete.	The study had no other sources of bias
8	Yang X J	2010	Intergroup allocation in the study was not randomize d.	The study explicitly stated that group concealment was not implemented.	No blinding was performed in the study.	The outcome measuremen t will not be affected.	No clear research proposal	Outcome data were complete.	The study had no other sources of bias
9	Zhao T C	2015	Intergroup allocation in the study was not randomize d.	The study explicitly stated that group concealment was not implemented.	No explicit reporting on whether subject blinding was implemented	The outcome measuremen t will not be affected.	A well-defined research proposal	Outcome data were complete.	The study had no other sources of bias

8. PRISMA 2020 Checklist

Section and Topic	Item #	Checklist item	Location where item is reported
**TITLE**			Y
Title	1	Identify the report as a systematic review.	Title
**ABSTRACT**			Y
Abstract	2	See the PRISMA 2020 for Abstracts checklist.	Abstract
**INTRODUCTION**			Y
Rationale	3	Describe the rationale for the review in the context of existing knowledge.	Introduction
Objectives	4	Provide an explicit statement of the objective(s) or question(s) the review addresses.	Introduction
**METHODS**			Y
Eligibility criteria	5	Specify the inclusion and exclusion criteria for the review and how studies were grouped for the syntheses.	Materials and Methods
Information sources	6	Specify all databases, registers, websites, organisations, reference lists and other sources searched or consulted to identify studies. Specify the date when each source was last searched or consulted.	Materials and Methods
Search strategy	7	Present the full search strategies for all databases, registers and websites, including any filters and limits used.	Materials and Methods
Selection process	8	Specify the methods used to decide whether a study met the inclusion criteria of the review, including how many reviewers screened each record and each report retrieved, whether they worked independently, and if applicable, details of automation tools used in the process.	Materials and Methods
Data collection process	9	Specify the methods used to collect data from reports, including how many reviewers collected data from each report, whether they worked independently, any processes for obtaining or confirming data from study investigators, and if applicable, details of automation tools used in the process.	Materials and Methods
Data items	10a	List and define all outcomes for which data were sought. Specify whether all results that were	Materials and Methods

Section and Topic	Item #	Checklist item	Location where item is reported
		compatible with each outcome domain in each study were sought (e.g. for all measures, time points, analyses), and if not, the methods used to decide which results to collect.	
	10b	List and define all other variables for which data were sought (e.g. participant and intervention characteristics, funding sources). Describe any assumptions made about any missing or unclear information.	Materials and Methods
Study risk of bias assessment	11	Specify the methods used to assess risk of bias in the included studies, including details of the tool(s) used, how many reviewers assessed each study and whether they worked independently, and if applicable, details of automation tools used in the process.	Materials and Methods
Effect measures	12	Specify for each outcome the effect measure(s) (e.g. risk ratio, mean difference) used in the synthesis or presentation of results.	Materials and Methods
Synthesis methods	13a	Describe the processes used to decide which studies were eligible for each synthesis (e.g. tabulating the study intervention characteristics and comparing against the planned groups for each synthesis (item #5)).	Results
	13b	Describe any methods required to prepare the data for presentation or synthesis, such as handling of missing summary statistics, or data conversions.	Materials and Methods
	13c	Describe any methods used to tabulate or visually display results of individual studies and syntheses.	Results
	13d	Describe any methods used to synthesize results and provide a rationale for the choice(s). If meta-analysis was performed, describe the model(s), method(s) to identify the presence and extent of statistical heterogeneity, and software package(s) used.	Materials and Methods
	13e	Describe any methods used to explore possible causes of heterogeneity among study results (e.g. subgroup analysis, meta-regression).	Materials and Methods
	13f	Describe any sensitivity analyses conducted to	Results

Section and Topic	Item #	Checklist item	Location where item is reported
		assess robustness of the synthesized results.	
Reporting bias assessment	14	Describe any methods used to assess risk of bias due to missing results in a synthesis (arising from reporting biases).	Materials and Methods
Certainty assessment	15	Describe any methods used to assess certainty (or confidence) in the body of evidence for an outcome.	Materials and Methods
**RESULTS**			Y
Study selection	16a	Describe the results of the search and selection process, from the number of records identified in the search to the number of studies included in the review, ideally using a flow diagram.	Results
	16b	Cite studies that might appear to meet the inclusion criteria, but which were excluded, and explain why they were excluded.	Results
Study characteristics	17	Cite each included study and present its characteristics.	Results
Risk of bias in studies	18	Present assessments of risk of bias for each included study.	Results
Results of individual studies	19	For all outcomes, present, for each study: (a) summary statistics for each group (where appropriate) and (b) an effect estimate and its precision (e.g. confidence/credible interval), ideally using structured tables or plots.	Discussion
Results of syntheses	20a	For each synthesis, briefly summarise the characteristics and risk of bias among contributing studies.	Results
	20b	Present results of all statistical syntheses conducted. If meta-analysis was done, present for each the summary estimate and its precision (e.g. confidence/credible interval) and measures of statistical heterogeneity. If comparing groups, describe the direction of the effect.	Results
	20c	Present results of all investigations of possible causes of heterogeneity among study results.	Results
	20d	Present results of all sensitivity analyses conducted to assess the robustness of the synthesized results.	Results

Section and Topic	Item #	Checklist item	Location where item is reported
Reporting biases	21	Present assessments of risk of bias due to missing results (arising from reporting biases) for each synthesis assessed.	Results
Certainty of evidence	22	Present assessments of certainty (or confidence) in the body of evidence for each outcome assessed.	Results
**DISCUSSION**			Y
Discussion	23a	Provide a general interpretation of the results in the context of other evidence.	Discussion
	23b	Discuss any limitations of the evidence included in the review.	Limitations
	23c	Discuss any limitations of the review processes used.	Limitations
	23d	Discuss implications of the results for practice, policy, and future research.	Discussion
**OTHER INFORMATION**			Y
Registration and protocol	24a	Provide registration information for the review, including register name and registration number, or state that the review was not registered.	Materials and Methods
	24b	Indicate where the review protocol can be accessed, or state that a protocol was not prepared.	Materials and Methods
	24c	Describe and explain any amendments to information provided at registration or in the protocol.	Materials and Methods
Support	25	Describe sources of financial or non-financial support for the review, and the role of the funders or sponsors in the review.	Support
Competing interests	26	Declare any competing interests of review authors.	Competing Interests
Availability of data, code and other materials	27	Report which of the following are publicly available and where they can be found: template data collection forms; data extracted from included studies; data used for all analyses; analytic code; any other materials used in the review.	Data Sources
